# Functional regulatory mechanism of smooth muscle cell-restricted *LMOD1* coronary artery disease locus

**DOI:** 10.1371/journal.pgen.1007755

**Published:** 2018-11-16

**Authors:** Vivek Nanda, Ting Wang, Milos Pjanic, Boxiang Liu, Trieu Nguyen, Ljubica Perisic Matic, Ulf Hedin, Simon Koplev, Lijiang Ma, Oscar Franzén, Arno Ruusalepp, Eric E. Schadt, Johan L. M. Björkegren, Stephen B. Montgomery, Michael P. Snyder, Thomas Quertermous, Nicholas J. Leeper, Clint L. Miller

**Affiliations:** 1 Department of Surgery, Division of Vascular Surgery, Stanford University School of Medicine, Stanford, California, United States of America; 2 Stanford Cardiovascular Institute, Stanford University School of Medicine, Stanford, California, United States of America; 3 Department of Genetics, Stanford University School of Medicine, Stanford, California, United States of America; 4 Department of Medicine, Division of Cardiovascular Medicine, Stanford University School of Medicine, Stanford, California, United States of America; 5 Department of Biology, Stanford University School of Medicine, Stanford, California, United States of America; 6 Department of Pathology, Stanford University School of Medicine, Stanford, California, United States of America; 7 Department of Molecular Medicine and Surgery, Karolinska Institutet, Stockholm, Sweden; 8 Department of Genetics and Genomic Sciences, Icahn Institute for Genomics and Multiscale Biology, Icahn School of Medicine at Mount Sinai, New York, United States of America; 9 Clinical Gene Networks AB, Stockholm, Sweden; 10 Department of Cardiac Surgery, Tartu University Hospital, Tartu, Estonia; 11 Department of Medical Biochemistry and Biophysics, Vascular Biology Unit, Karolinska Institutet, Stockholm, Sweden; 12 Department of Physiology, Institute of Biomedicine and Translation Medicine, University of Tartu, Tartu, Estonia; 13 Center for Public Health Genomics, Department of Public Health Sciences, Department of Biochemistry and Molecular Genetics, and Department of Biomedical Engineering, University of Virginia, Charlottesville, Virginia, United States of America; University of Pennsylvania, UNITED STATES

## Abstract

Recent genome-wide association studies (GWAS) have identified multiple new loci which appear to alter coronary artery disease (CAD) risk via arterial wall-specific mechanisms. One of the annotated genes encodes *LMOD1* (Leiomodin 1), a member of the actin filament nucleator family that is highly enriched in smooth muscle-containing tissues such as the artery wall. However, it is still unknown whether *LMOD1* is the causal gene at this locus and also how the associated variants alter LMOD1 expression/function and CAD risk. Using epigenomic profiling we recently identified a non-coding regulatory variant, rs34091558, which is in tight linkage disequilibrium (LD) with the lead CAD GWAS variant, rs2820315. Herein we demonstrate through expression quantitative trait loci (eQTL) and statistical fine-mapping in GTEx, STARNET, and human coronary artery smooth muscle cell (HCASMC) datasets, rs34091558 is the top regulatory variant for *LMOD1* in vascular tissues. Position weight matrix (PWM) analyses identify the protective allele rs34091558-TA to form a conserved Forkhead box O3 (FOXO3) binding motif, which is disrupted by the risk allele rs34091558-A. FOXO3 chromatin immunoprecipitation and reporter assays show reduced FOXO3 binding and *LMOD1* transcriptional activity by the risk allele, consistent with effects of *FOXO3* downregulation on *LMOD1*. *LMOD1* knockdown results in increased proliferation and migration and decreased cell contraction in HCASMC, and immunostaining in atherosclerotic lesions in the SMC lineage tracing reporter mouse support a key role for LMOD1 in maintaining the differentiated SMC phenotype. These results provide compelling functional evidence that genetic variation is associated with dysregulated LMOD1 expression/function in SMCs, together contributing to the heritable risk for CAD.

## Author summary

It is now estimated that nearly ~50% of one’s lifetime risk for coronary artery disease is genetically predetermined. While genetic studies have led to the identification of several novel disease risk factors, the mechanisms driving many of these associations are yet to be fully identified. Here, we take advantage of a recent finding identifying genetic variation in a smooth muscle cell restricted gene called Leiomodin1, and investigate the causal gene and mechanism by which this risk factor predisposes individuals to coronary artery disease. By implementing several complementary approaches, we extend previous findings demonstrating that this genetic variant is associated with attenuated expression of Leiomodin 1. Interestingly, we demonstrate that this association is attributed to altered binding of a transcription factor Forkhead Box 3. More importantly, we show that reduced Leiomodin 1 results in phenotypic modulation of smooth muscle cells, a process related to diseases such as atherosclerosis. Interestingly, Leiomodin 1 levels were found to be reduced in smooth muscle cells within atherosclerotic lesions relative to those around the fibrous cap. These studies provide new insights into the heritable mechanisms of coronary artery disease with Leiomodin 1 as a potential therapeutic target.

## Introduction

Coronary artery disease (CAD) is the leading cause of mortality worldwide, and poses considerable public health burden despite current treatment and lifestyle interventions. Both genetic and environmental factors contribute to CAD susceptibility, however early detection and prevention requires a deeper understanding of the primary causes of the disease process itself. Recent meta-analyses of genome-wide association studies (GWAS) have identified 161 genetic loci associated with CAD at genome-wide significance[1–6]. Importantly, around 60% of these loci have been associated independently of classical risk factors[7], and given previous functional evidence of candidate causal genes at these loci[8–11], suggests an involvement of pathways intrinsic to the vessel wall. This is supported by the recent CAD GWAS meta-analysis, which combined targeted genotyped, multi-ethnic individuals with individuals from the CARDIoGRAMplusC4D consortium, to identify 15 new loci, within or near genes having arterial wall-specific functions[4].

Interestingly, one of these new loci (lead SNP rs2820315) is found to reside in a smooth muscle cell (SMC)-restricted gene annotated as *Leiomodin 1* (*LMOD1*)[4]. LMOD1 is a member of the LMOD family of proteins, known to bear close resemblance to actin capping proteins called Tropomodulins (TMODs). However, unlike TMODs, only LMODs possess an extended C terminus containing an additional WH2 domain (~17aa actin monomer motif) [12,13]. While LMOD2 and LMOD3 are found to be primarily restricted to skeletal and cardiac muscles, we and others have previously demonstrated LMOD1 is highly enriched in smooth muscle containing tissues, including main arteries and visceral organs of the gastrointestinal system[14–17]. Furthermore, of the three LMODs, only *LMOD1* is identified as a specific downstream target of the SMC master regulator serum response factor (SRF)-Myocardin (MYOCD) complex[15]. More recently, a homozygous nonsense mutation in *LMOD1* has been linked to a rare congenital visceral myopathy called megacystis microcolon intestinal hypoperistalsis syndrome (MMIHS)[18]. Detailed analyses performed using *Lmod1* deficient mice, revealed this phenotype to be a consequence of disrupted actin filament assembly, cytoskeletal arrangement and impaired SMC contractility[18]. Interestingly, this defect is reportedly analogous to the thin-filament disorganization and nemaline myopathy observed in mice and humans with *Lmod3* deficiency[19]. In addition, our recent epigenomic profiling study of human coronary artery smooth muscle cells (HCASMC) has led to the identification of a novel CAD associated regulatory variant, rs34091558, in the *LMOD1* locus[20]. Although we determined this variant resides in a region of accessible chromatin defined by the Assay for Transpose Accessible Chromatin (ATAC-seq) and is marked by the H3K27ac active enhancer histone modification, the underlying mechanism for this association remains unknown.

Herein, we provide confirmatory evidence that rs2820315, near *LMOD1*, is a CAD-specific GWAS association, which co-localized with the expression quantitative trait locus (eQTL) association in both GTEx and STARNET artery tissues, and that rs34091558 (in tight LD with rs2820315) is a likely causal variant at this locus. We investigate the genetic regulatory mechanism using a combination of *in silico*, *in vitro* and *in vivo* approaches in primary HCASMC and human and animal disease models, which highlight a role for LMOD1 in maintaining the differentiated SMC phenotype. Collectively these findings are expected to improve our understanding of the ‘hidden risk’ for CAD, and further close the gap between disease-associated regulatory variation and relevant vascular wall phenotypes.

## Results

### *LMOD1* is associated with CAD but not traditional risk factors

**Fig 1:**
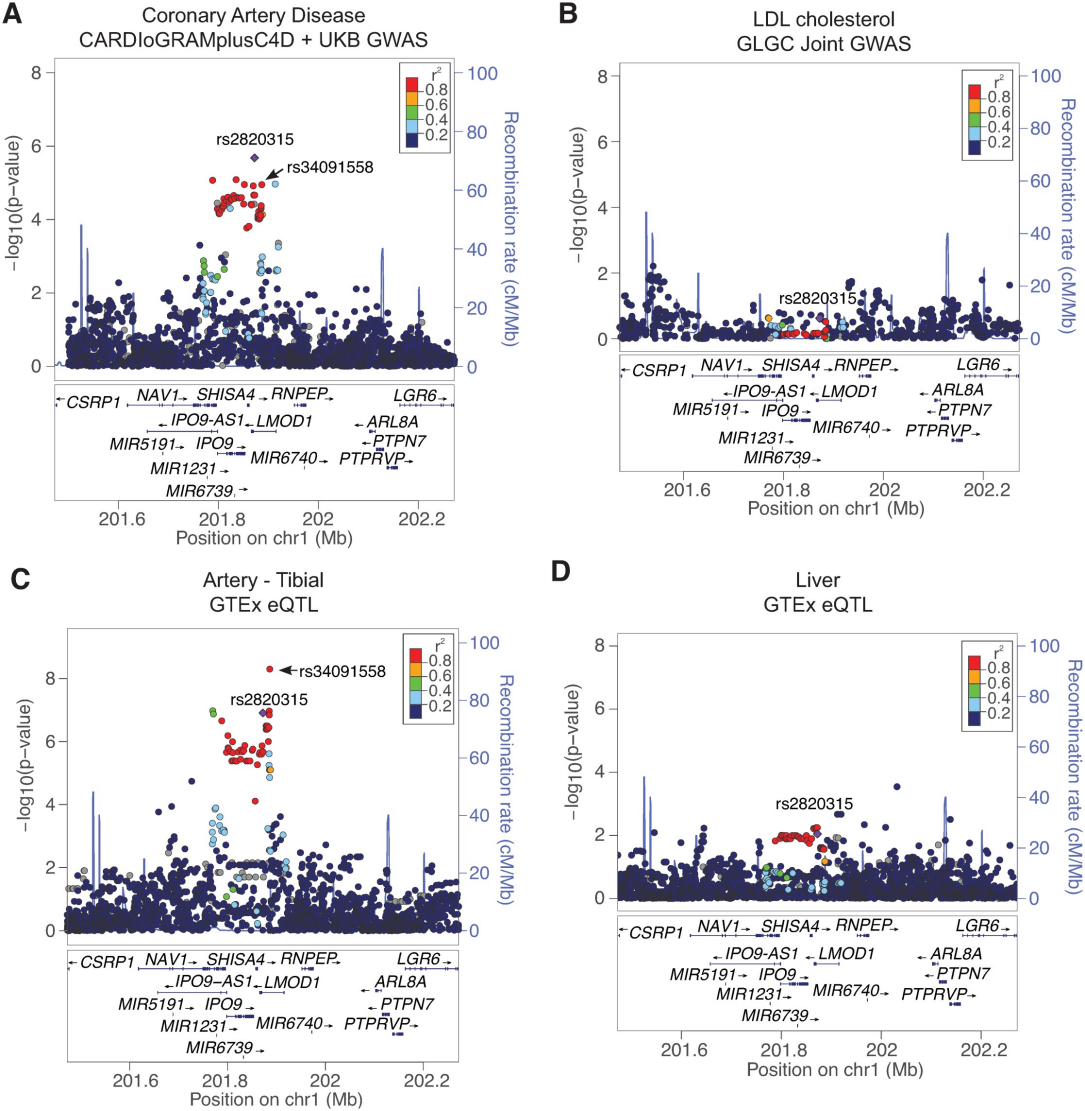
Association of rs2820315 with CAD and *LMOD1* expression. LocusZoom plot depicting association results at rs2820315 locus at chromosome 1q32.1 in (A) CAD from CARDIoGRAMplusC4D + UKB GWAS, (B) LDL cholesterol from Global Lipid Genetics Consortium and rs2820315 eQTL association (highlighting LD SNP rs34091558) with *LMOD1* and other candidate genes in (C) Tibial Artery and (D) Liver in the GTEx dataset. Circles represent SNPs associated using an additive or recessive model, color-coded for LD (r^2^) with the lead GWAS SNP rs2820315 (purple diamond), which resides near the *LMOD1* gene.

The *LMOD1* locus was previously associated with CAD (lead SNP rs2820315; *P* = 7.70E-10) in a meta-analysis (88,192 CAD cases and 162,544 controls) of *de novo* genotyped multi-ethnic cohorts combined with previous genotyped CARDIoGRAMplusC4D cohorts in a study that identified 15 loci related to arterial wall mechanisms[4]. To follow up on these findings, we mapped the lead risk variant, rs2820315, in the latest CARDIOGRAMplusC4D and UK Biobank meta-analysis for CAD (10,801 CAD cases inclusive of angina and 137,371 controls)[5] and identified rs2820315 associated at sub genome-wide significance (*P* = 2.09E-06, Fig 1A), consistent with previously published reports[4]. Interestingly, rs2820315 was not associated with low-density lipoprotein (LDL) cholesterol (Fig 1B) in large-scale studies such as the Global Lipids GWAS Consortium[21]. Similarly, we did not detect a significant association for rs2820315 with other CAD risk factors such as fasting glucose, fasting insulin, systolic blood pressure and low vWF in previous GWAS datasets from MAGIC[22], in the International Consortium for Blood Pressure (ICBP) [23], and CHARGE[24] (S1 Fig). These results extend previous findings implicating the *LMOD1* locus in the ‘hidden’ heritable risk for CAD, potentially through regulation of vessel wall processes.

### *LMOD1* expression is enriched in artery tissues and eQTL colocalizes with GWAS signal

**Fig 2:**
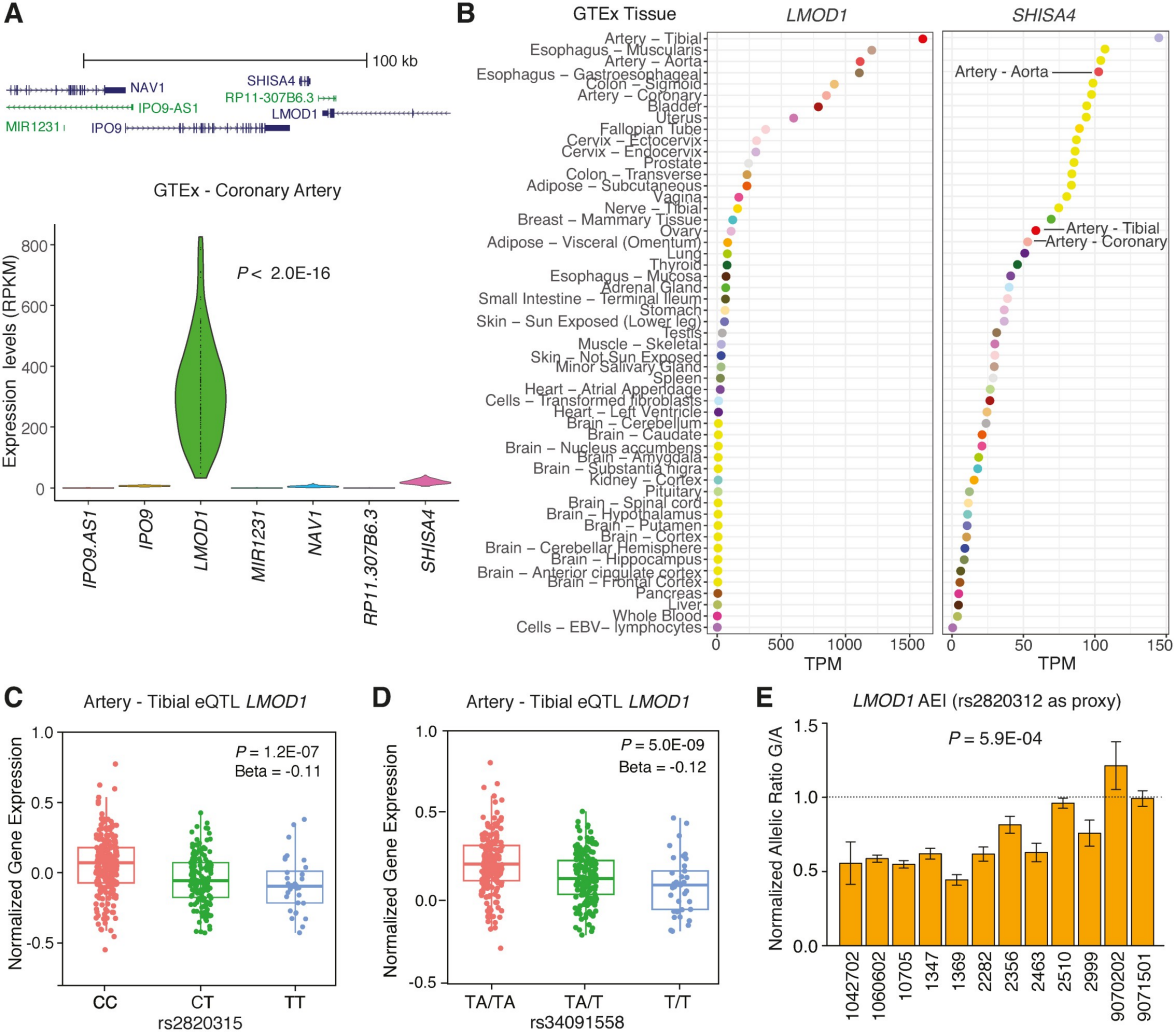
Validation of *LMOD1* as a causal gene in GTEx database. (A) Schematic indicating neighboring genes in the *LMOD1* locus using RefSeq database, coding transcripts in blue and non-coding transcripts in green (upper panel). Expression profile of coding and non-coding transcripts at *LMOD1* locus in coronary artery tissues (n = 133) from GTEx database (v6p) (lower panel). (B) Tissue expression profile of *LMOD1* and *SHISA4* across the entire GTEx dataset (v7) ranked according to median transcripts per million (TPM) for each tissue. Boxplots showing rs2820315 allele dosage correlated with *LMOD1* (C) and rs34091558 allele dosage correlated with *LMOD1* (D) in tibial artery tissue in GTEx dataset (v7). (E) Allelic expression imbalance (AEI) for *LMOD1* in HCASMCs heterozygous at rs2820315 and rs34091558, using rs2820312 coding SNP as a proxy (n = 12). Values represent mean ± standard deviation of triplicates of cDNA/gRNA normalized allelic ratios.

The majority of published GWAS results report the nearest gene(s) to the lead SNP, however recent findings from the multi-tissue Genotype-Tissue Expression (GTEx) project[25] demonstrate that this is correct for only 40% of GWAS signals (21 traits tested)[26]. Therefore, we evaluated eQTL results in GTEx to identify potential causal genes at the *LMOD1* locus. Using such an approach, we identified a significant eQTL association of rs34091558 with *LMOD1* (*P* = 5.0E-09; r^2^ = 0.94 with lead GWAS SNP rs2820315 in Europeans) in tibial artery tissue (Fig 1C), but not in liver tissue (Fig 1D). We also identified significant eQTLs for variants associated with *LMOD1* in SMC containing tissues in GTEx, such as lung (S2A and S2B Fig) and esophagus mucosa (S2C and S2D Fig). Importantly, we only observed GWAS overlapping eQTLs in artery GTEx tissues for variants associated with *LMOD1 (P* = 5.0E-09*)*, and not nearby genes: *NAV1*, *MIR1231*, *IPO9-AS1*, *IPO9*, *SHISA4*, *RP11-307B6*.*3* (Fig 1C, S1 Table). Thus, we queried the relative expression of all genes at the *LMOD1* locus (Fig 2A, upper panel) in atherosclerosis prone tissues such as human coronary artery in the GTEx dataset. We observed abundant *LMOD1* expression in this tissue type, however the expression of neighboring genes was barely detectable (Fig 2A, lower panel). A similar expression pattern was observed in other atherosclerosis prone and SMC-enriched tissues such as aorta, and tibial artery in GTEx (S3A Fig). As expected little or no *LMOD1* expression was detected in liver (S3A Fig). Similarly, we evaluated relative gene expression in the STARNET datasets and observed highest expression of *LMOD1* in normal mammary artery (MAM) and atherosclerotic aortic root (AOR) compared to liver (S3B Fig). We then ranked candidate gene expression in all GTEx tissues (normalized by transcripts per million) and observed *LMOD1* is highly ranked in both artery and SMC-enriched tissues than compared to neighboring genes *SHISA4*, *IPO9* and *NAV1* (Figs 2B and S4). These results were consistent with the direction of the rs2820315-T and rs34091558-T risk alleles associated with lower *LMOD1* expression in tibial arteries (*P* = 1.2E-07 and 5.0E-09, respectively) in the GTEx (Fig 2C and 2D). Despite the high expression of *LMOD1* in coronary artery tissue, eQTLs were undetectable, potentially due to the lower sample number and greater tissue heterogeneity in coronary arteries relative to tibial arteries (www.gtexportal.org). Using summary-based mendelian randomization (SMR) colocalization analysis of CARDIoGRAMplusC4D GWAS and multi-tissue GTEx and STARNET *cis*-eQTL summary statistics[27], we observed *LMOD1* to be the top candidate gene at this locus (P_SMR_ = 7.25E-05, P_HET_ = 0.137). Taken together, these data strongly implicate *LMOD1* as a causal gene at this new CAD locus.

To further validate *cis*-acting effects on *LMOD1* gene expression, we measured allelic expression imbalance (AEI) in cultured HCASMCs using the coding SNP, rs2820312, as a proxy (r^2^ = 0.83 with lead SNP rs2820315; S5A Fig) and detected an approximate 0.5–0.8 fold imbalance of the reference allele (G) over the variant allele (A) in 11 out of 12 heterozygous samples (P = 5.9E-0.4) using TaqMan based quantification of cDNA/gDNA (Figs 2E and S5B). We extended our findings by performing pyrosequencing based allelic quantification and observed a similar trend (S5C Fig). Together these data are consistent with the direction of the eQTL results reported above (Fig 2C) and suggest that *LMOD1* levels are decreased through a *cis*-regulatory mechanism in subjects carrying the risk variant.

### Fine-mapping *LMOD1* locus in HCASMC

To investigate the regulatory landscape at the *LMOD1* locus and prioritize potential causal variants responsible for attenuated *LMOD1* expression, we leveraged the Roadmap Epigenomics dataset comprising 98 different primary cells and tissues. Having previously identified rs34091558 (in tight LD with the lead SNP rs2820315), to reside in a region of open chromatin and H3K27ac marked active enhancer in HCASMCs[20], we thus focused on the H3K27ac mark across several CAD related, SMC-enriched tissues, as well as other primary tissues. Consistent with the GTEx RNA-seq tissue distribution, we observed rs34091558 to reside in an H3K27ac marked region in aorta and SMC-enriched gastrointestinal tissues (S6A Fig). Interestingly, this region also displayed high specificity in HCASMCs when compared to other ENCODE cell datasets (S6B Fig). We also analyzed the 15 chromatin state ChromHMM Roadmap datasets, and found that rs34091558 preferentially resides in active enhancer states in these SMC-enriched tissues, consistent with activation rather than repression of gene expression (S7 Fig). To further test whether chromatin annotations are sufficient to delineate causal variants at this locus we implemented probabilistic fine-mapping approaches such as the Probabilistic Annotation INTegratOR (PAINTOR)[28] and Probabilistic Identification of Causal SNPs (PICS)[29] algorithms, based on HCASMC functional annotations, haplotype structure and summary GWAS results. As expected, the lead SNP rs2820315 was the top ranked variant using the LD-based PICS, but rs34091558 had the highest posterior probability using PAINTOR (S2 and S3 Tables). We augmented these results using an exhaustive shotgun stochastic search algorithm (FINEMAP) to identify causal configurations from GWAS and eQTL variants[30], and again observed rs34091558 with the highest posterior probability (S4 Table). This is consistent with our findings on rs34091558 being the only CAD risk variant that overlapped a conserved sequence and multiple functional annotations in HCASMC (S5 Table). This is also consistent with conditional analysis results of rs34091558 on the latest CAD GWAS association results at the *LMOD1* locus (S8 Fig). While it remains possible that multiple causal variants may exist under specific regulatory contexts, these fine-mapping results strongly indicate that rs34091558 is a top candidate causal variant at this locus.

### Predicted FOXO3 binding and regulation of *LMOD1* at rs34091558

**Fig 3:**
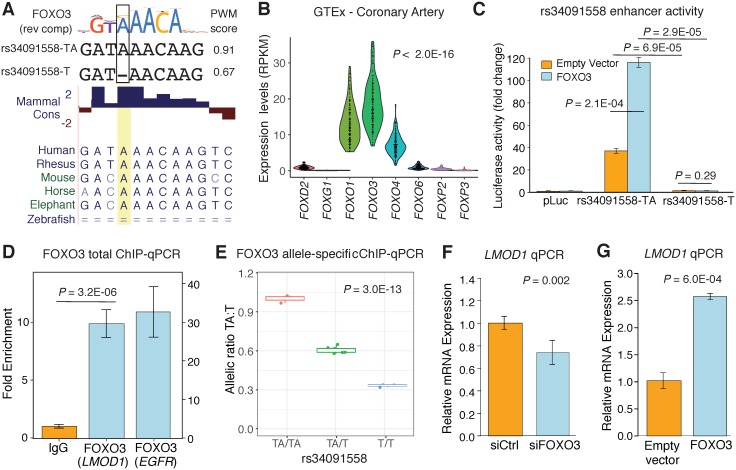
Functional mapping of the *LMOD1* locus identifies FOXO3 dependent mechanism at rs34091558. (A) Prediction of rs34091558 non-risk (TA) and risk allele (T) on FOXO3 binding based on JASPAR PWM scores. Human sequence (hg38) is shown aligned to the consensus FOXO3 sequence and mammalian genomic sequences and PhyloP conservation track. (B) Relative expression (RPKM) of candidate Forkhead transcription factors in coronary artery tissues (n = 133) from the GTEx dataset. (C) Luciferase activity examined for each of the *LMOD1* enhancer constructs in A7r5 SMCs in the presence of *FOXO3*. Results were reproduced in n = 3 independent experiments performed in quadruplicates. (D) Chromatin immunoprecipitation (ChIP) assay for *LMOD1* and *EGFR* (as a positive control region) in HCASMC chromatin lysates immunoprecipitated with antibodies to FOXO3 or a negative control rabbit IgG. Results were repeated in n = 3 independent studies. (E) Allele specific ChIP (haploChIP) for FOXO3 protein in DNA derived from cultured HCASMC ChIP experiments. DNA from cell line homozygous for the ancestral allele was used as a positive control and arbitrarily set to 1. Values represent mean ± standard deviation of triplicates. Similar results were observed from n = 4 independent lines for each genotype. (F) Quantitative RT-PCR analysis of *LMOD1* in HCASMCs following knock down of endogenous *FOXO3* (F) or overexpression of *FOXO3* (G). The results were reproduced in n = 3 independent experiments.

Given these findings we then pursued the potential molecular mechanism(s) linking the rs34019558 risk variant (single nucleotide deletion) to reduced *LMOD1* expression. To this end, we scanned the sequence containing rs34091558-TA and rs34091558-T for putative transcription factor binding sites (TFBS) using a combination of *in silico* prediction tools, including JASPAR[31], MATCH[32], HOCOMOCO[33] and HaploReg[34]. Interestingly, we identified that the ancestral protective allele (rs34091558-TA) is predicted to bind a mammalian conserved family of transcription factors annotated as Forkhead box (FOX), while the derived risk allele (rs34091558-T) is predicted to significantly disrupt this binding (JASPAR PWM scores 0.91 > 0.67) (Fig 3A and S6 Table). RNA-seq analysis of all candidate FOX transcription factors in GTEx datasets, identified *FOXO3* as highly expressed in coronary, tibial, and aorta artery tissues in comparison to other FOX family members (P < 2.2E-16) (Figs 3B and S9) and in a manner similar to that of *LMOD1*, thus revealing FOXO3 as a potential regulator of *LMOD1*.

In order to evaluate the impact of rs34091558 on FOXO3 protein binding and activity, we utilized enhancer luciferase reporter constructs containing either the protective or risk allele [20], driven by a minimal promoter, and thereafter tested the response of each construct to an expression plasmid encoding human FOXO3 in cultured A7r5 SMCs. As predicted, we detected significant basal allelic effects with the ancestral protective allele (rs34091558-TA) having greater enhancer activity than the risk allele, rs34091558-T (Fig 3C, *P* = 6.9E-05). These results provide independent confirmation of our previous findings [20]. Importantly, we observed even more pronounced allelic differences in reporter activity upon FOXO3 overexpression (Fig 3C, *P* = 2.9E-05). Given that FOXO3 strongly activated the protective allele but not the risk allele, suggests that FOXO3 mediated *trans*-activation is partly responsible for these allelic effects.

Given the known limitations of episomal based reporter assays, we then performed FOXO3 chromatin immunoprecipitation (ChIP) in cultured HCASMCs to investigate the impact of rs34091558 in the native chromatin context. Using such an approach, we first demonstrated significant enrichment of FOXO3 protein at the rs34091558 enhancer region (*P* = 3.25E-06) (Fig 3D), following which we measured the allele-specific effects of rs34091558 on FOXO3 binding using both homozygous and heterozygous HCASMC donors at rs34091558. In agreement with the reporter assays and eQTL direction of effect above, we observed the ancestral protective allele, rs34092558-TA, to preferentially bind FOXO3 protein relative to the derived risk allele rs34091558-T (*P* = 3.0E-13) (Fig 3E). These results suggest that carriers of the risk allele have reduced *LMOD1* expression in part due to the inhibition of FOXO3 protein binding at rs34091558.

To assess whether FOXO3 specifically regulates endogenous *LMOD1* expression, we first transfected HCASMCs with a small interfering RNA (siRNA) to *FOXO3* and then measured *LMOD1* gene expression after 24 hours. In addition to observing reduced *FOXO3* expression (S10A and S10C Fig), cells transfected with si*FOXO3* also had reduced *LMOD1* mRNA expression (Fig 3F). Extending these findings, we overexpressed FOXO3 in A7r5 SMCs (S10B Fig), and observed a consistent increase in *LMOD1* expression in these cells (Fig 3G).

### PDGF-BB reduces LMOD1 expression and FOXO3 mediated transcription at rs34091558

**Fig 4:**
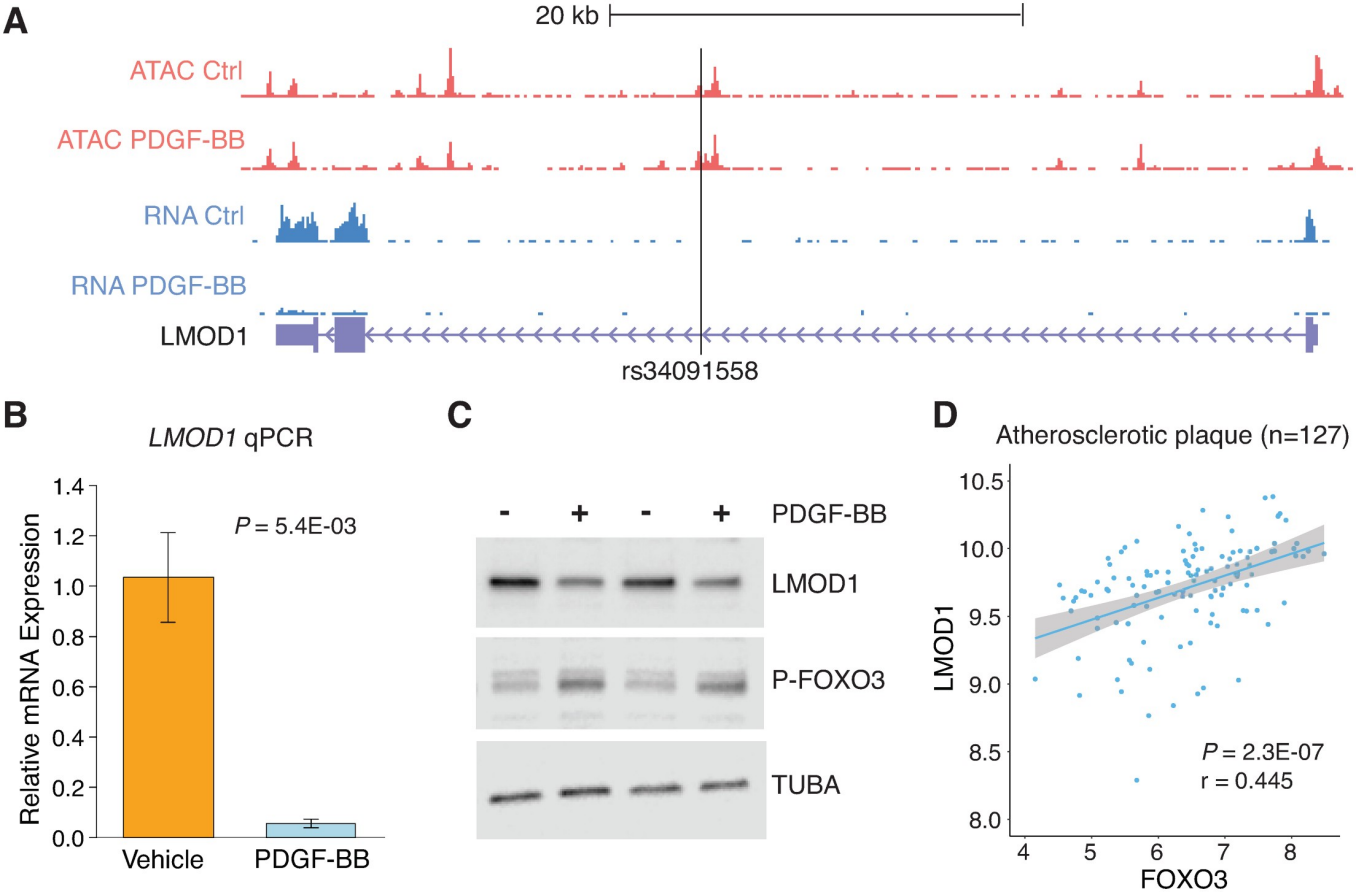
*LMOD1* expression is mediated by PDGF-BB-FOXO3 signaling cascade. (A) UCSC Browser screenshot of the *LMOD1* CAD locus at chromosome 1q32.1 highlighting the candidate causal variant, rs34091558, overlapping ATAC-seq open chromatin and RNA-seq tracks in HCASMCs treated with PDGF-BB (n = 2 biological replicates). Genomic coordinates refer to hg19 assembly. Quantitative RT-PCR (B) and Western blotting (C) data revealing PDGF-BB and phosphorylated-FOXO3 (P-FOXO3) mediated LMOD1 expression. (D) Co-expression microarray analysis performed in a cohort of carotid atherosclerotic plaques (n = 127) indicating that *LMOD1* and *FOXO3* are positively correlated in arterial tissues.

To begin exploring the relevant upstream signals responsible for FOXO3 regulation of *LMOD1*, we treated HCASMC with various growth factors and cytokines known to influence SMC differentiation. Silencing of transforming growth factor beta (*TGFB1*), a known pro-differentiation cytokine did not alter *LMOD1* expression in HCASMC (S11 Fig). However, treatment with platelet derived growth factor BB (PDGF-BB) an established mitogenic anti-differentiation growth factor[35], significantly downregulated *LMOD1* mRNA levels via qRT-PCR (P = 0.0054), consistent with previously shown HCASMC RNA-seq results [20,36] (Fig 4A and 4B). These confirmatory results support that *LMOD1* expression is regulated by PDGF-BB signaling and preferentially associated with the differentiated phenotype in HCASMC. We extend these findings by Western blotting, which revealed attenuated LMOD1 protein levels but increased phosphorylated FOXO3 levels in response to PDGF-BB (Fig 4C). Increased phosphorylated FOXO3 downstream of PDGF-BB signaling has been shown to limit the transcriptionally active FOXO3 protein[37]. Previous findings have demonstrated significant differential expression of *LMOD1* in human atherosclerotic carotid artery plaque microarray data (n = 127) from the Biobank of Karolinska Endarectomies (BiKE) study[36]. Using this dataset we identified a strong correlation of *LMOD1* and *FOXO3* transcripts (Fig 4D), which suggests that LMOD1 and FOXO3 may be co-regulated during atherosclerosis *in vivo*.

### *LMOD1* deficiency impairs SMC proliferation, migration and contraction

**Fig 5:**
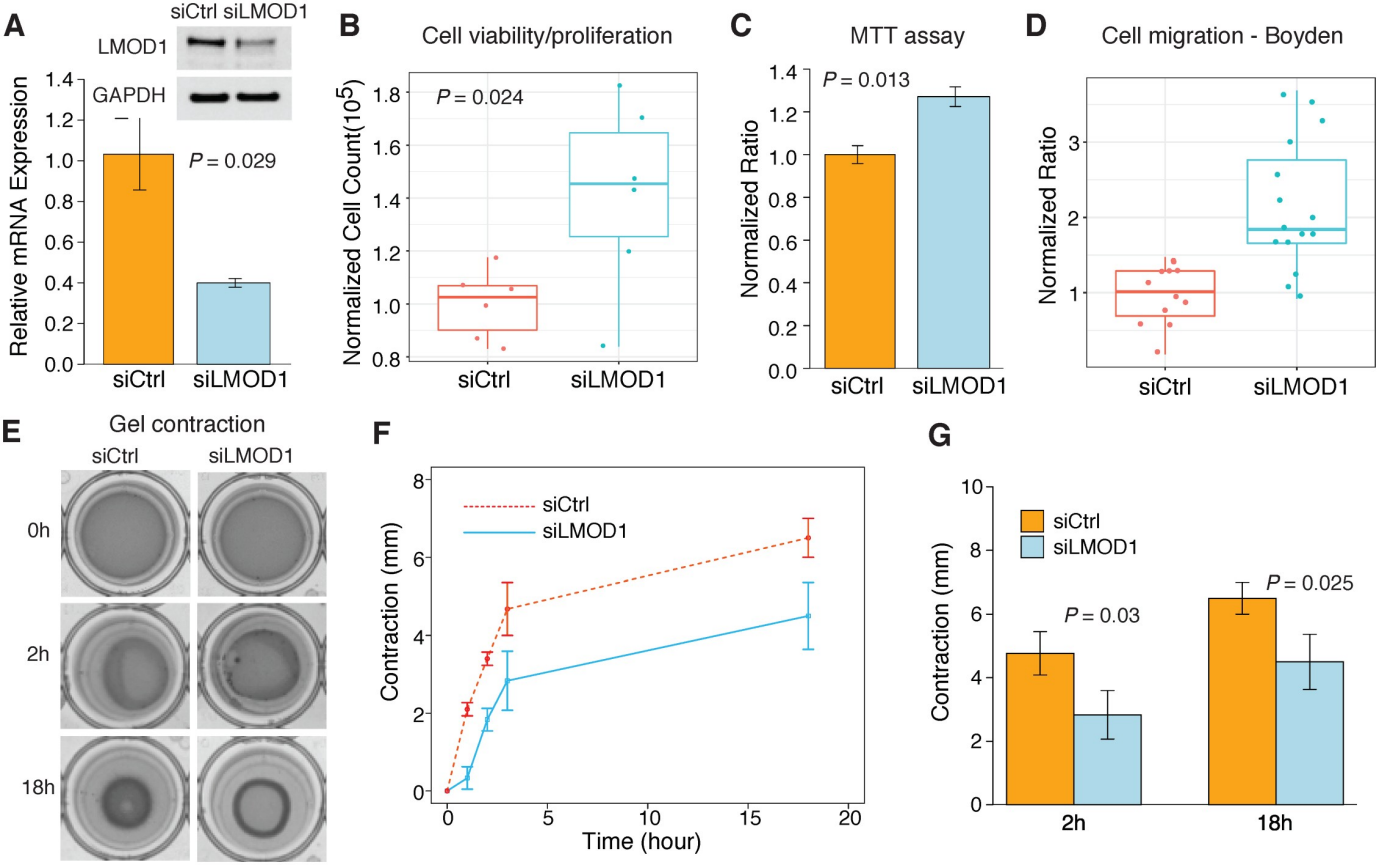
LMOD1 deficiency promotes de-differentiated SMC phenotype. (A) Quantitative RT-PCR and Western blotting analysis confirming endogenous knockdown of LMOD1 in cultured HCASMCs. Cell proliferation measured in HCASMCs transfected with siCtrl or si*LMOD1* via (B) trypan blue exclusion and (C) CellTiter96 Non-Radioactive Cell Proliferation Assay (MTT assay). (D) Differences in cell migration assessed via Boyden Chamber assay in HCASMCs transfected with siCtrl or si*LMOD1*. (E) Images of cell contraction in siCtrl or si*LMOD1* transfected HCASMCs at indicated time points and represented via quantification (F-G). All data represent mean ± standard deviation from n = 3 independent experiments.

Having confirmed RNAi mediated *LMOD1* knockdown at the mRNA and protein levels (Fig 5A) we next performed a series of functional assays to interrogate the effects of reduced *LMOD1* expression on SMC differentiation. Compared to control siRNA, *LMOD1* siRNA transfected cells displayed a significantly higher proliferation rate as measured using trypan blue cell viability/proliferation assays (*P* = 0.024) (Fig 5B) and CellTiter96 (Promega) cell proliferation assays (*P* = 0.013) (Fig 5C). Also, *LMOD1* deficient HCASMCs were associated with increased migration in Boyden chamber assays (*P* = 1.4E-04) (Fig 5D). In keeping with these findings, cell contraction assays revealed that loss of *LMOD1* significantly repressed HCASMC contraction on collagen gel substrate, which was maintained for 18 hours (Fig 5E–5G). This effect on contraction is consistent with previous results in intestinal SMCs[38]. These data suggest that LMOD1 is essential to preserving the contractile phenotype observed in differentiated SMCs and its loss potentiates SMCs to adopt a de-differentiated phenotype often associated with CAD.

### LMOD1 localization in the SMC lineage tracing mouse during atherosclerosis

**Fig 6:**
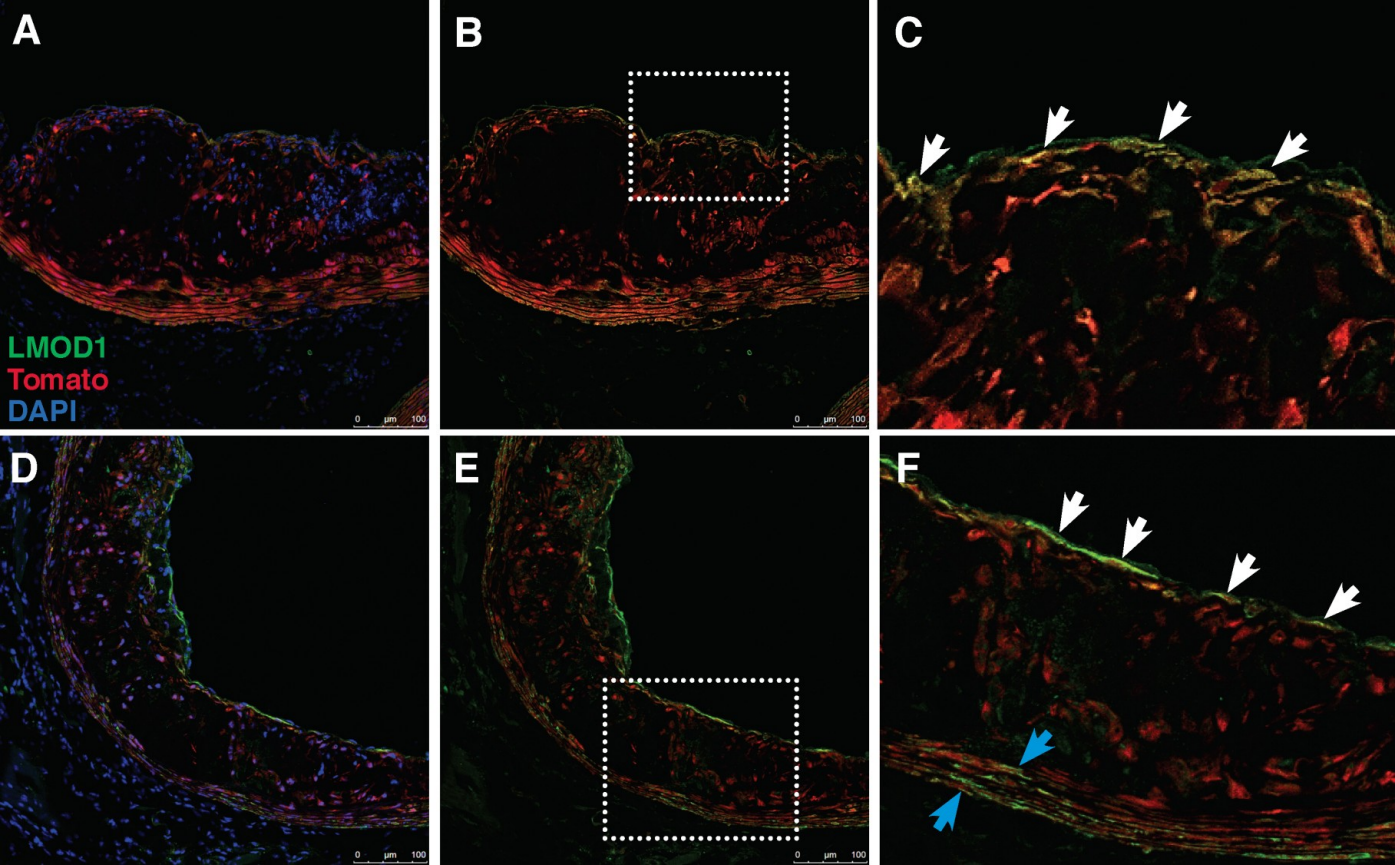
LMOD1 expressing cells associate with atherosclerotic fibrous cap. (A and D) Immunofluorescence staining for LMOD1, Tomato and DAPI in the brachiocephalic artery of 24 week old *Myh11*-*Cre-ER*^T2^ *ROSA26-STOP*-tdTomato *Apoe*^-/-^ mice fed high fat diet for 18 weeks. (B and C) LMOD1 and tomato expressing cells were identified in the fibrous cap (white arrows). (E and F) Another lesion showing LMOD1 and tomato expressing cells in the media (blue arrows). Tomato positive cells in the lesion stained negative for LMOD1 in the lesion. Images were captured using a 20x objective and are representative of n = 3 independent mice.

SMCs have been shown to play critical roles in both the expansion of the intima (largely comprised of proliferative SMCs) in early stages of atherosclerosis and stabilizing the developing lesion in later stages through the formation of a thick fibrous cap (comprised of differentiated SMCs) [39]. While previous reports demonstrated differential staining of LMOD1 in carotid atherosclerosis [36], it was still not clear what type of SMCs are implicated. This is important given that SMCs have heterogeneous responses during atherosclerosis and may elicit detrimental or protective effects depending on the nature of their phenotypic transitions to a de-differentiated state [40,41]. To validate our *in vitro* findings and determine the potential role of SMC-specific LMOD1 in atherosclerosis, using a previously authenticated LMOD1 antibody [42], we tracked LMOD1 expression in atherosclerotic lesions in the brachiocephalic artery of the SMC-specific lineage tracing mouse *Myh11*-*Cre-ER*^T2^ *ROSA26-STOP*^Flox^ tdTomato *Apoe*^-/-^ [40] after 18 weeks of high-fat diet and observed that LMOD1 co-localizes with tdTomato-positive cells of SMC-origin in both the media and fibrous cap outside the lesion (Fig 6A–6C). In contrast, LMOD1 was largely absent from tdTomato-positive cells within the lesion, which mostly consist of de-differentiated/proliferative SMCs[40]. We observed similar results in independent mouse lesions and noted that LMOD1 was highly expressed along the thin fibrous cap and throughout the media (Fig 6D–6F), suggesting that LMOD1, as a differentiation SMC marker gene, may participate in plaque stability. This is consistent with previous immunohistochemistry results showing a loss of LMOD1-expressing cells in carotid atherosclerotic lesions [36], and refines the localization of LMOD1 in specific SMC-derived populations during disease. These data also suggest that LMOD1 downregulation may represent a critical step in SMC phenotypic transitions during atherosclerosis, while maintained LMOD1 expression in differentiated SMC may be beneficial.

**Fig 7:**
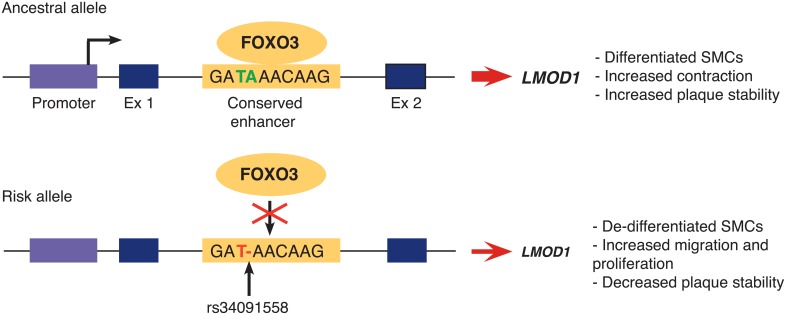
Proposed mechanism for how rs34091558 impairs FOXO3 mediated *LMOD1* expression therefore altering CAD risk. Individuals having the rs34091558-TA ancestral, protective allele would be at reduced CAD risk due to greater *LMOD1* expression levels, whereas individuals having the rs34091558-T derived, risk allele would be at greater CAD risk due to reduced *LMOD1* expression levels, through a FOXO3-dependent mechanism.

Taken together our working model (Fig 7) demonstrates that individuals carrying the protective allele, rs34091558-TA have elevated *LMOD1* expression levels due to increased FOXO3 binding, promoting SMCs to adopt a differentiated phenotype, a hallmark of stable atherosclerotic lesions thought to reduce CAD risk. In contrast, individuals carrying the risk allele, rs34091558-A have reduced *LMOD1* levels due to the inhibition of FOXO3 binding, resulting in SMCs adopting a proliferative phenotype, a hallmark of unstable lesions and increased disease risk.

## Discussion

In this study we performed a comprehensive functional analysis of a SMC-restricted gene associated with CAD. By combining large-scale regulatory annotations and fine-mapping with mechanistic perturbations and functional assays in primary HCASMCs, we confirm previous studies and reveal new insights into the causal CAD mechanisms operating in the vessel wall. In particular, this integrative approach identified a critical role for the rs34091558 deletion risk variant in regulating FOXO3 mediated transcription of *LMOD1*. This regulation of *LMOD1* was strongly antagonized by the well-established mitogenic PDGF-BB signaling pathway, consistent with previous reports and provides a physiological link in coronary SMCs. Further, by employing loss-of-function assays in HCASMC and immunostaining of atherosclerotic lesions *in vivo*, we identified a role for LMOD1 in controlling the differentiated SMC phenotype and potentially regulating plaque stability. These findings are expected to shed light on the understudied heritable risk associated with CAD and may inform the development of more precise therapeutic interventions in future investigations.

To date, 161 loci have been identified for CAD from large-scale genome wide association studies, with the majority of these associations being independent of traditional risk factors[3,5,6]. Previous studies have focused on systems based approaches to categorize and prioritize these loci into regulatory networks and functional pathways for future follow up[43–45]. Fifteen loci, within or near genes having arterial wall-specific functions were recently reported[4]. Of these, 5 loci are found to reside near genes preferentially expressed in SMCs (*LMOD1*, *SERPINH1*, *DDX59*–*CAMSAP2*, *TNS1*, *PECAM1*). *SERPINH1* (rs590121 lead SNP) is predicted to increase risk of hemorrhage; *DDX59*-*CAMSAP2* (rs1867624) may be involved abnormal vascular development; PECAM1 (rs1867624) may regulate vascular inflammation and endothelial dysfunction; and *LMOD1* (rs2820315) is implicated in SMC differentiation[4]. In our previous study we also discovered regulatory variants at seven candidate CAD loci, including 9p21.3, *SMAD3*, *PDGFD*, *IL6R*, *BMP1*, *CCDC97*/*TGFB1* and *LMOD1*, that associated with enhancer epigenetic marks in HCASMC[20]. However, *LMOD1* is the only gene that appears to have a SMC-restricted expression pattern as a SRF/MYOCD target gene. Given that LMOD1 is a member of the LMOD family of actin filament nucleators[12], it may potentially play a critical role in regulating smooth muscle actin filament nucleation and function. This is supported by the recent identification of a recessive mutation in a premature stop codon of *LMOD1* as a cause of a rare congenital visceral myopathy, MMIHS[18], which usually arises due to autosomal dominant mutations in the smooth muscle enriched actin gamma 2 (ACTG2) gene. The identification of common regulatory variation in *LMOD1* associated with CAD supports the notion that a genetic dosage effect exists for Mendelian and complex diseases involving smooth muscle.

Here, we confirm that the lead associated variant at the *LMOD1* locus, rs2820315, is also the lead SNP in multiple CAD cohorts including the most recent UKBiobank cohort comprising ~500,000 individuals. We also show that this variant is an eQTL for *LMOD1*, with the risk allele associated with lower *LMOD1* expression levels, in SMC tissues including tibial artery from the GTEx dataset. Fine-mapping and expression analyses demonstrated that *LMOD1* is the most likely causal gene at this locus, which is enriched in vascular tissues, and allelic imbalance assays in HCASMC suggest a *cis*-regulatory mechanism of *LMOD1*. Pinpointing causal variants at a given locus is often challenged by the strong LD between the lead risk variant and hundreds of candidate regulatory variants[46]. Many of these variants reside in regions of accessible chromatin, and are occupied by transcription factors or histone modification. Using ATAC-seq and ChIP-seq molecular profiling in HCASMC we previously identified a top candidate regulatory variant at this locus, rs34091558 (r^2^ = 0.94 with rs2820315), that resides in a region of open chromatin, TF binding, and enhancer activity. In fact, this was the only such variant identified at the *LMOD1* locus (S4 Table). Here, we provide more definitive statistical fine-mapping evidence supporting its causality and identify this variant to disrupt binding of the FOXO transcription factor family, particularly FOXO3, given its endogenous expression in HCASMC and vascular tissues. It remains possible that other variants (despite lack of complete functional annotation) contribute to the regulation of *LMOD1*. This is in agreement with a ‘multiple enhancer variant’ model for complex diseases[47], in which multiple risk alleles in the same haplotype could contribute regulatory effects on *LMOD1* through cooperative or independent mechanisms. Also, it is important to note that this proposed mechanism probably relates to European and Asian populations where the LD is generally high (r2~0.9) but not in African populations, where due to higher recombination rates these two SNPs (rs34091558 and rs2920315) are not in LD.

The mechanistic link of FOXO3-*LMOD1* is intriguing given that FOXO3 has been associated with body mass index and adiposity[48,49] and may explain the strong eQTL association of rs2820315 with *LMOD1* in adipose tissues. Interestingly *FOXO3* was found to be the most highly expressed of the *FOXO* family members, particularly in artery tissues. Previous evidence suggests that FOXO transcription factors can regulate tissue-specific expression either directly or by heterodimerizing with cell-specific cofactors[50]. Here, we also identified an enrichment of FOXO3 motifs in HCASMC-specific open chromatin peaks compared to other tissues (S12 Fig), supporting a potential tissue-specific binding preference in SMCs. Previous studies have observed altered FOXO3 expression and phosphorylation with PDGF-BB and IGF mediated signaling in dermal fibroblasts[37]. We demonstrate here that LMOD1 protein levels are significantly down-regulated with PDGF-BB stimulation, while phosphorylated FOXO3 (P-FOXO3) levels are upregulated. This supports a potential mechanism by which PDGF-BB mediated P-FOXO3 sequesters available nuclear FOXO3 for binding to this enhancer, thus resulting in reduced *LMOD1*. FOXO3 binding sites were also identified at the promoter of *LMOD1*, thus it remains possible that mechanisms independent of this enhancer could partially contribute to the PDGF-BB mediated effects.

Our functional assays in HCASMC with *LMOD1* gene silencing support a role for this gene in regulating the differentiated SMC phenotype, consistent with previous reports in intestinal SMCs[18]. *LMOD1* downregulation resulted in increased SMC proliferation, viability, migration, and decreased contraction. Further, using an established SMC lineage tracing mouse bred on the *Apoe*-null background with 16 weeks of high-fat diet challenge, we localized LMOD1 to the forming fibrous cap in the brachiocephalic artery. Given the direction of the human genetic association studies and prior expression analyses in carotid arteries[36], these results suggest that LMOD1 may serve a protective role in the setting of atherosclerosis, and potentially stabilize the developing lesion. Supporting this ‘atheroprotective SMC’ hypothesis, LMOD1 was also localized to the medial layer of the lesion. While LMOD1 appears to selectively mark differentiated SMCs *in vivo*, the functional effects of LMOD1 deficiency should also be validated *in vivo* using various models of atherosclerosis progression. Future studies using single-cell transcriptomic profiling in lineage-traced SMCs during atherosclerosis may further define the causal functional role of LMOD1 as a key regulator of SMC transformation during the disease process.

## Materials and methods

### Tissue distribution and eQTL mapping in GTEx and STARNET

Association between lead variant, rs2820315, and candidate regulatory variant, rs34091558, with *LMOD1* gene expression was determined by querying the public eQTL database from the Genotype-Tissue Expression (GTEx) project and the Stockholm-Tartu Atherosclerosis Reverse Engineering Task (STARNET)[51] project, as previously described[20]. Briefly, both rsIDs and candidate genes in the entire locus were queried in all tissues in GTEx and STARNET and results represented as -log_10_p-values across a ~1Mb region in LocusZoom. Note that rs34091558 is misannotated in GTEx as rs397789675 and can be found based on its chromosome:position ID, chr1:201886769 (hg19). Alternatively, dosage plots of normalized gene expression levels relative to variant genotype are shown for both GTEx and STARNET eQTLs. For comparing the tissue distribution of candidate gene expression levels, reads per kilobase per million mapped reads (RPKM) or transcripts per million (TPM) values were extracted from the GTEx databases. Similarly, EDAseq normalized reads were extracted from the STARNET database (atherosclerotic aortic root, internal mammary artery, and liver), as previously described [20].

### Conditional analysis of *LMOD1* association using CAD GWAS summary data

Conditional analysis of rs34091558 or rs2820315 SNP association with CAD was performed using the Genome-wide Complex Trait Analysis (GCTA) software package, GCTA-COJO[52]. Briefly, summary statistics data from the latest CARDIoGRAMplusC4D and UK Biobank meta-analysis was downloaded from the consortium website. A 1Mb region flanking lead SNP rs2820315 was extracted using subset function and data was reformatted in R according to the input file requirements. Either rs34091558 or rs2820315 was added to snp file cond.snplist and cojo was run using the default parameters: gcta64—bfile test—cojo-file test.ma—cojo-cond cond.snplist—out test.

### Epigenomic mapping using Roadmap, ENCODE, and HCASMC datasets

Tissue-specific regulatory mapping of *LMOD1* variants was assessed using genome-wide epigenomic data in the Roadmap Epigenomics[53] and Encyclopedia of DNA Elements (ENCODE)[54] projects. Cardiovascular tissues, smooth muscle enriched tissues and non-smooth muscle tissues were selected and queries were performed using the rsID for regulatory variants in the locus and ChIP-seq data for the active enhancer histone modification H3K27ac. Chromatin states from the 15-state ‘core’ model using Roadmap histone modification ChIP-seq data were queried using ChromHMM[55] and visualized in the Roadmap Epigenomics browser. HCASMC epigenomic profiles generated from custom ATAC-seq and ChIP-seq (H3K27ac, H3K4me1, and H3K4me3) were queried for each variant as previously described[20].

### Probabilistic fine-mapping using GWAS summary statistics and HCASMC functional annotations

Statistical fine-mapping of the *LMOD1* locus was first conducted using the Probabilistic Identification of Causal SNPs (PICS)[56]. Given the observed pattern of the association at the *LMOD1* locus, using CARDIoGRAMplusC4D and UKBiobank meta-analysis summary statistics and variant coverage using Phase 3 1000 Genomes Project data, 10,000 permutations were simulated with the association of the lead SNP rs2820315 fixed and all possible associations obtained from the LD SNPs. Based on these statistical fluctuations the probabilities of LD SNPs being the causal variant were then estimated using Bayes’ theorem. The test makes the assumption that the prior probability of each SNP to be a causal variant or the lead SNP rs2820315 is equal. Also, probabilities were calculated for all SNPs in LD at r^2^ >0.5 with the lead SNP.

Fine-mapping analysis was also conducted using the Probabilistic Annotator INTegratOR (PAINTOR v3.0)[28] implemented on the command line. Briefly, summary statistics from the latest CARDIoGRAMplusC4D and UK Biobank meta-analysis were integrated with LD data by generating a matrix with pairwise Pearson correlations for each SNP, and a matrix containing binary formatted HCASMC functional annotation data (ATAC-seq and ChIP-seq for H3K27ac, H3K4me1, H3K4me3) for SNPs overlapping a given annotation. Functional annotations were used as prior probabilities and learned from the data via Empirical Bayes to prioritize causal variants (without explicit assumptions on maximum number of causal variants, *j*). Candidate causal variants were estimated by posterior probabilities and observed association Z-scores using a standard multivariate normal assumption model, where *C*_*j*_ is the causal vector, *λ*_*j*_ is the non-centrality parameter, and *Σ*_*j*_ is the LD matrix of pairwise correlation coefficients:

$$P(C_{j};\lambda_{j})=N(Z_{j};\Sigma_{j}(\lambda_{j}\circ C_{j}),\Sigma_{j})$$

### Colocalizaton fine-mapping using GWAS summary statistics and eQTL datasets

Colocalization fine-mapping of candidate target genes in the *LMOD1* locus was performed using Summary-based Mendelian Randomization (SMR)[27] by integrating the latest CARDIoGRAMplusC4D and UK Biobank GWAS summary statistics with GTEx and STARNET eQTL summary data. We tested the pleiotropic effect of all *cis*-eQTLs associated with both gene expression at the *LMOD1* locus and CAD phenotype. We considered all *cis*-eQTL associations at P < 5E-05 and GWAS associations at P < 0.05. We used genetic data from individuals of European ancestry from 1000 Genomes Phase 3 as the reference panel. We also performed the HEterogeneity In Dependent Instruments (HEIDI) test to determine whether the GWAS and eQTL associations are likely driven by the same variant (not significant for HEIDI test). Therefore we considered GWAS target genes at a threshold of P_SMR_ < 0.05 and P_HEIDI_ > 0.05.

Statistical fine-mapping of variants in the *LMOD1* locus was performed using both GWAS and relevant eQTL summary statistics in FINEMAP v1.0, an exhaustive Shotgun Stochastic Search (SSS) algorithm implemented on the command line[30]. Like other fine-mapping tools, PICS, PAINTOR, and CAVIARBF, FINEMAP uses Bayes’ theorem to estimate a posterior probability of causal configurations. The most computationally efficient method to compute the unnormalized posterior using the Bayes factor (BF) was used, where *γ* is the maximum likelihood estimate (MLE) of the causal SNP effects, *y* is the mean-centered values for a quantitative trait, *X* is the SNP genotype matrix, *m* is the maximum number of causal configurations, *k* is the number of causal configurations, and *p*_*k*_ is the prior probability for each causal configuration:

$$p_{2}^{*}(y,X)={\left(\frac{m}{k}\right)}^{-1}p_{k}\times BF(\gamma :NULL)$$

The shotgun stochastic search of FINEMAP allowed more causal configurations to be considered, without the need to fix the number of causal SNPs.

### Cell culture

Commercially available HCASMCs purchased from Lonza or Cell Applications were cultured in SMC basal medium (SmBM) supplemented with SmGM-2 SingleQuots kit as previously described[20]. SMC integrity was established by reports of positive smooth muscle α actin staining and negative von Willebrand (factor VIII) performed by the suppliers. Rat aortic SMCs (A7r5) were maintained in DMEM containing high glucose and 10% fetal bovine serum (Thermo Fisher Scientific) without antibiotics or antimycotics as previously described[15]. For PDGF-BB stimulation experiments, HCASMCs were seeded at ~70% confluency in 6 well dishes. Once attached, cells were serum starved for 24 hours and thereafter treated with either vehicle or PDGF-BB (20ng/ml). Following a 24-hour incubation, RNA or protein was isolated from cells.

### Allelic expression imbalance (AEI)

Genomic DNA and RNA were prepared from individual HCASMC donor lots determined to be either heterozygous or homozygous genotypes at proxy SNP rs2820312 (determined by whole genome sequencing). Genomic DNA and total RNA were isolated using the Qiagen DNeasy Blood and Tissue kit and the Qiagen miRNeasy Mini kit, respectively, according to the manufacturer’s instructions. Total cDNA was prepared from 1 μg RNA using the High Capacity cDNA Reverse Transcription kit (Applied Biosystems). Each qPCR reaction contained 20ng Genomic DNA or 20ng cDNA, 40x rs2820312 TaqMan SNP genotyping probe (Applied Biosystems), and 2x Universal Master Mix (Applied Biosystems). The PCR cycling conditions were as follows: an initial denaturation at 95 °C for 10 min, followed by 50 cycles of 95 °C for 10 sec and 60 °C for 45 sec. AEI was determined using the TaqMan SNP genotyping probe for rs2820312 (S7 Table) and expressed as the normalized allelic ratio of cDNA/gDNA. Calibration of the SNP genotyping assay was determined by mixing 20 ng of gDNA or cDNA, homozygous for each allele at the following ratios: 4:1, 2: 1 1:1, 1:2, 1: 4. The Log2 ratio of the VIC/FAM intensity from gDNA at cycle 50 was then plotted against the Log ratio of the two alleles to generate a linear regression standard curve. The Log ratio of VIC/FAM intensity of cDNA was fitted to the standard curve for the correction. These values were then normalized to the ratio of gDNA for each allele to obtain the normalized allelic ratio.

### Pyrosequencing

Pyrosequencing assays were performed by the Protein And Nucleic acid (PAN) facility (Stanford) as previously described[57] with assays designed using PyroMark Assay Design software (Qiagen). 20 ng gDNA or cDNA was amplified using forward and reverse pyrosequencing primers under the following conditions: 94°C 4 min, (94°C 30 sec, 60°C 30 sec, 68°C 45 sec) ×35, 68°C 6 min and PCR products were verified by gel electrophoresis. Pyrosequencing reaction was performed on PCR reactions using PyroMark Q24 (Qiagen) according to manufacturer’s instructions. Allelic quantitation was obtained automatically from the mean allele frequencies derived from the peak heights using PyroMark Q24 software.

### RNA Isolation, cDNA generation and quantitative RT-PCR

Total RNA was isolated from cell lines using the miRNeasy mini kit (Qiagen) as per the manufacturer’s instructions and subsequently quantified using a nanodrop (Thermo Fisher Scientific). cDNA was generated using the iScript cDNA synthesis kit (Bio-Rad) or High Capacity cDNA Reverse Transcription Kit (Thermo Fisher Scientific). Differences in mRNA expression were examined and quantified using TaqMan based real time PCR (Applied Biosystem ViiA^™^7). Data generated was normalized to *GAPDH*, converted to relative quantity values using the 2^-ddCt^ method and expressed as the relative fold change. Detailed information of the probes used to assess expression of target genes are listed in S7 Table.

### siRNA studies and overexpression studies

HCASMC were seeded at ~70% confluency in 6-well dishes containing medium supplemented with serum and growth factors. Once attached, cells were serum starved (treated with medium containing only 0.2% serum) overnight and transfected the following day with either 10nM negative control (si*Ctrl*, Catalog # 4390846) or siRNA to *FOXO3* (si*FOXO3*, Catalog # s2843) using Lipofectamine RNAiMAX (Life Technologies) as per the manufacturer’s instructions. 24 h post transfection, RNA was isolated from these cells for measuring mRNA expression. In the case of overexpression studies, cDNA generated from HCASMC derived RNA and enzyme-clamped primers (S7 Table) were used to PCR amplify a 2021 bp fragment encompassing the human FOXO3 coding sequence. The resulting PCR product was cloned into the EcoRV-HindIII site of the pCMV-Tag2B plasmid (Agilent). The authenticity of the plasmid generated was confirmed via Sanger sequencing. Confluent A7r5 cells were transfected with either 1ug pCMVTag2B or FOXO3-pCMV-Tag2B using Lipofectamine 2000 (Life Technologies) as per the manufacturer’s instructions. 4–6 hours post transfection, cells were re-fed with fresh growth medium and total RNA was isolated after 48 hours.

### Enhancer luciferase reporter assays

*LMOD1* enhancer reporters were constructed by first identifying the putative enhancer sequence based on HCASMC ATAC-seq and H3K27ac flanked region at chr1: 201886639–201886794 (hg19). 160 nt single stranded oligonucleotide sequences (S7 Table) containing either the protective rs34091558-TA allele or the risk rs34091558-T allele and cloning site were annealed and the resulting dsDNA oligos were subcloned into the pLuc-MCS (Agilent) luciferase reporter vector driven by a minimal promoter. All reporter constructs were validated by Sanger sequencing. A7r5 SMCs were plated at ~70% confluency in 24 well-dishes and co-transfected with either luciferase reporter, *Renilla* luciferase internal control plasmid, and human *FOXO3* or empty vector expression plasmids on the following day using Lipofectamine 3000, as per the manufacturer’s instructions (Thermo Fisher Scientific). Cells were lysed 48 h later in passive lysis buffer and analyzed on a luminometer (Molecular Devices) using the Dual-Luciferase Reporter Assay System (Promega) as per the manufacturer’s instructions. All transfections were performed in quadruplicate and were repeated at least three times. Results are presented as the fold change of the ratio of firefly to *Renilla* luciferase relative light units (RLU) with the control condition (pLuc + Empty vector) set to 1.0.

### FOXO3 motif density in HCASMC-specific ATAC-seq regions

HCASMC and other tissue-specific open chromatin peaks were selected using the most stringent criteria, i.e. the peak can only appear in the selected tissue. This yielded 24372 specific peaks for brain, 7332 for HCASMC, 6388 for lung fibroblasts, 11249 for heart. Density plots were constructed using ChIP-Cor Analysis Module Feature Correlation Tool v1.5.3. Input range was defined as +/- 10000 bp from the centered reference features. Window width was set to 500 bp and counts cut-off value was set to 999999. Normalization was global, i.e. histogram entries were normalized by the total number of reference and target feature counts and the window width. The output graph represents target feature abundance as fold change relative to a genome average.

FOXO3 PWM matrix was taken from JASPAR CORE vertebrates 2018 database. PWMScan—Genome-wide position weight matrix (PWM) scanner was used to scan GRCh37/h19 version of the human genome using the following parameters: p-value cut-off 0.0001, background base composition 0.29,0.21,0.21,0.29, search strand—both, non-overlapping matches—on. To confirm that enrichment of FOXO3 PWM near HCASMC-specific open chromatin peaks does not occur due to the correlation in base compositions of FOXO3 PWM and HCASMC specific open chromatin regions, we repeated the analysis using randomized FOXO3 PWM. Using such defined parameters, we obtained 712104 FOXO3 PWM binding site genome-wide and after randomizing the FOXO3 matrix we obtained 740737 FOXO3-randomized sites and performed correlation with ChIP-Cor Analysis Module as described above.

### ChIP and allele-specific ChIP–qPCR

ChIP assays were performed as previously described[58]. Briefly, 2x10^7^ HCASMCs were crossed linked using 1% formaldehyde and quenched with glycine. Nuclear lysates were prepared using cold hypotonic buffer, followed by lysis in 1X RIPA buffer (Millipore). Chromatin nuclear lysates were then sheared to fragments of 100–500 bp using a Bioruptor Pico sonicator (Diagenode) at constant duty cycle, output control 3–4, 20 sec pulses for 16 rounds. Equal amounts of anti-rabbit IgG (Dako), FOXO3 (Novus Biologicals, Cat # NBP2-16521), and JUNB (Santa Cruz, Cat# sc-73) antibody were added to sheared chromatin to immunoprecipitate TF–DNA complexes and incubated overnight at 4°C. Dynabeads Protein G (Life Technologies) were used to capture the antibody–TF–DNA complexes and followed by washing with ice-cold RIPA buffer and PBS and eluted twice in 1X TE buffer containing 1% SDS or 0.67% SDS for 10 min each at 65 °C. The crosslinked Protein–DNA complex were reversed overnight at 65 °C and ChIP DNA was recovered using Qiagen PCR Purification kits according to the manufacturer’s instructions. For ChIP-qPCR experiments, primers spanning the FOXO3 binding site within the *LMOD1* enhancer and *EGFR* control regions were used. qPCRs were performed using SYBR green chemistry (Applied Biosystems). Primer information is listed in S7 Table. In the case of allele-specific assays, DNA from ChIP experiments and a customized TaqMan probe were used for performing qPCR. Homozygous cell line for the risk allele was used as a positive control and the mean ratio of major allele/minor allele (FAM/VIC) at 50 cycles were normalized to input DNA ratios.

### Protein extraction and Western blotting

Total protein was isolated from cultured cell lines using 1X RIPA buffer (Millipore) supplemented with 1X Halt Protease Inhibitor Single-Use Cocktail (Thermo Fisher Scientific) as previously described[59]. Protein concentration for all samples was determined using Pierce BCA Protein Assay Kit (Thermo Fisher Scientific). Equal amounts of protein samples were loaded and separated on precast gels (Bio-Rad) and thereafter transferred onto PVDF membranes (Bio-Rad). Following a 1-hr incubation of these membranes in 5% skim milk solution prepared in 1X TBST, membranes were probed with commercially available antibodies recognizing LMOD1 (Proteintech, 1:1000), p-FOXO3 (Cell Signaling, 1:500) and alpha-tubulin (TUBA, Cell Signaling, 1:1000) overnight at 4°C. Membranes were subsequently rinsed with TBST and incubated with appropriately matching HRP conjugated goat anti rabbit (1:3000) secondary antibody for 1 hour at room temperature. Upon completion, membranes were briefly incubated with Pierce ECL Western Blotting Substrate (Thermo Fisher Scientific) after which protein expression was detected and captured using the Li-Cor odyssey imaging system.

### Cell viability assay

Cell viability was determined by implementing previously described Trypan Blue assays[60]. In short, equal number of transfected SMCs were plated in a 6-well dish and incubated overnight. Once attached, cells were serum starved for 48 hours and thereafter incubated with complete growth medium. 24 hours later cells were trypsinized, and briefly incubated with Trypan Blue, following which cells were manually counted using a hemocytometer. Alternatively, cell viability was also assessed by conducting a CellTiter96 Cell Proliferation Assay/MTT Assay (Promega) according to the manufacturer’s instructions. Briefly, transfected serum starved cells treated with complete growth medium for 24 hours, were incubated with 15 μl /well of Dye Solution for 4 hours at 37°C and thereafter with Solubilization Solution/Stop mix for 1 hour. Fluorescence was recorded at 570/650 nm using a plate reader (SpectraMax 190, Molecular Devices).

### Boyden chamber migration assay

SMC migration was assessed by performing standard Boyden chamber assays, as previously described[61]. Briefly, transfected SMCs were serum starved for 48 hours following which they were trypsinized and counted such that equal number of cells were distributed onto the upper chamber of 8.0 μm transwell migration chambers (BD Biosciences). Following a 24-hour incubation, cells that migrated into the lower chamber were fixed in cold methanol, stained with hematoxylin, imaged using a Leica inverted microscope and manually counted in a blinded manner.

### Cell contraction assay

6x10^5^ HCASMCs were transfected with either scrambled siRNA or *siLMOD1*. Following 24 hours of transfection, cells were trypsinized and used for a collagen based cell contraction assay according to manufacturer’s instruction (Cell Biolabs, San Diego, CA). Briefly, a mixture of cold Collagen Gel Working Solution (collagen solution: PBS: Neutralization Solution = 28: 7: 1) and 0.1 ml 2x10^5^ cell suspension was incubated in a 24-well dish at 37°C for optimum polymerization. Following an hour of incubation, cell culture medium was added atop of each collagen lattice and cells were incubated in a 37°C, 5% CO2 chamber. After 48 hours, cell contraction was initiated by gently releasing collagen gels from the sides of the culture dishes with a sterile spatula and changes in collagen gel size were measured by a ruler at different time points: 0 hr, 1 hr, 3 hr, 6 hr, and 18 hr.

### Mouse atherosclerosis lineage tracing models

Animal protocols were approved by the Stanford University Administrative Panel on Laboratory Animal Care. *ROSA26-STOP*^flox^*tdT*^+/+^, *Myh11*-*CreER*^T2^ and *Apoe*^-/-^ and were used in this study. To begin, we crossed *ROSA26-STOP*^flox^*tdT*^+/+^ with *Myh11-CreER*^T2^ to generate *ROSA26-STOP*^flox^*tdT*^+/+^; *Myh11-CreER*^T2^ mice. Since the *Myh11*-*CreER*^T2^ transgene is located on the Y chromosome, male *ROSA26-STOP*^flox^*tdT*^+/+^; *Myh11*-*CreER*^T2^ were bred to female *Apoe*^-/-^ to generate a cohort of *ROSA26-STOP*^flox^*tdT*^+/+^; *Myh11-CreER*^T2^; *Apoe*^-/-^ mice. In order to activate the *Cre*-recombinase, 5–6 week old male *ROSA26-STOP*^flox^*tdT*^+/+^; *Myh11*-*CreER*^T2^; *Apoe*^-/-^ mice were administered tamoxifen daily through intraperitoneal injections for 5 consecutive days. Upon completion, experimental mice were fed a high-fat (Western-type) diet, containing 21% anhydrous milk fat and 0.15% cholesterol (Dyets) for 18 weeks following which the brachiocephalic arteries from mice were harvested, rinsed with PBS, fixed with 4% paraformaldehyde and embedded in OCT (Sakura) and sectioned at 7 μm thickness using a cryotome (CM1950, Leica).

### Immunofluorescence

For detecting LMOD1 expression within the atherosclerotic lesion, brachiocephalic artery tissue sections were stained with a primary antibody specific to LMOD1 (Proteintech, 1:400) as previously described[18]. Briefly, tissue sections were rehydrated with PBS, permeabilized with 0.1% Triton X-100 and thereafter incubated in serum free protein blocking buffer (DAKO) for 1 hour. Upon completion, tissue sections were incubated overnight at 4°C with LMOD1 antibody, briefly rinsed with PBS after which they were incubated in goat anti-rabbit secondary antibody (A21245, Thermo Fisher Scientific) for 1 hour and ultimately mounted with medium containing DAPI (Sigma, F6057). Images were captured with a Leica SP8 inverted confocal microscope.

### Human atherosclerotic carotid artery microarray expression analysis

Human atherosclerotic carotid artery lesions were obtained from patients undergoing endarterectomy surgery for stable (asymptomatic) (n = 40) or unstable (symptomatic) (n = 87) carotid stenosis, as part of the Biobank of Karolinska Endarterectomies (BiKE)[62]. Normal control arterial samples (n = 10) were obtained from the iliac and radial arteries from healthy organ donors without any history of cardiovascular disease. Briefly, tissue was snap frozen in liquid nitrogen before pulverizing to a fine powder using a pre-chilled mortar and pestle, then resuspended in Qiazol lysis reagent (Qiagen) and homogenized with a rotor stator tissue homogenizer. Total RNA was extracted as described above using the miRNeasy Mini Kit (Qiagen) and RNA quality assessed using a Bioanalyzer 2100 (Agilent). Global gene expression profiles were analyzed by Affymetrix HG-U133 plus 2.0 Genechip microarrays from 127 patient derived plaque samples and 10 donor control samples. Robust multi-array average (RMA) normalization was performed and processed gene expression data presented in Log_2_ scale. These processed microarray data are available under Gene Expression Omnibus (GEO) accession number GSE21545. The raw data is also available through a formal application process by contacting the study authors directly.

### STARNET gene expression and eQTL biobank and data processing

Gene expression and genotyping data from the STARNET database[20], were obtained from atherosclerotic aortic root (AOR), internal mammary artery (MAM) and liver from up to 600 CAD patients (determined eligible for the study and consented by the ethical committees of the Karolinska Institutet and Tartu University) that were obtained during coronary artery bypass graft open-heart surgeries. GenomeWideSNP_6 arrays (Affymetrix) were used for genotyping gDNA. Total RNA was isolated from the atherosclerotic arterial wall or internal mammary artery. Gene expression levels were determined using a standard RNA-seq library preparation and sequencing protocol (Illumina HiSeq 2500), followed by normalization of raw read counts to adjust for library size and batch effects. Briefly, samples with <1 million reads were removed, and genes with <1 counts per million in more than 50% of the samples were also removed. EDAseq was then used to normalize the library size and GC % content, and outliers were removed based on a gender-expression test, covariates were adjusted with linear regression, outliers were again removed and last rank quantile normalization was performed. ‘Normalized Expression Counts’ represent counts before rank quantile normalization. Adjusted read counts were subsequently log_2_-transformed, and the association between genotype and expression was tested using a linear model. eQTLs were called using the MatrixEQTL R package running a linear model and returning all calls with a maximum *P* value of 0.05. A follow-up conditional analysis was done to test the *P*(max SNP | SNP) as well as *P*(SNP | max SNP) for each gene. This was calculated by regressing the genotype of SNP_1 onto the residuals of SNP_2 regressed onto the gene expression, where SNP_2 is the SNP being conditioned on (for example, *P*(SNP_1 | SNP_2)).

### Ethics statement

All samples reported in this study were obtained with approval of the Institutional Review Board at the respective institutions under written informed consent. Samples collected in the STARNET study were obtained from patients determined eligible for the study and consented by the ethical committees of the Karolinska Institutet and Tartu University. All atherosclerotic carotid plaque and donor control samples collected from the Biobank of Karolinska Endarterectomies (BiKE) were obtained with informed consent from patients, organ donors or their guardians. The BiKE study is approved by the Ethical Committee of Northern Stockholm (approval numbers 02/147 and 2009/295-31/2).

### Statistical analyses

Experiments were performed using at least three independent preparations and individual treatments/conditions performed in triplicate or quadruplicate unless otherwise noted. Sample sizes for individual experiments were determined based on power calculations to detect small effects in cultured cells/tissues. All data are presented as mean + standard deviation of replicates using RStudio for statistical analysis. In experiments requiring comparisons across two groups, standard unpaired *t*-tests with Welch’s correction were implemented for determining significance using RStudio. In the case of more than 2 groups, a one-way analysis of variance (ANOVA) followed by Bonferroni’s post-hoc test for individual significance was performed using RStudio.

### Web resources

CARDIoGRAMplusC4D Consortium (http://cardiogramplusc4d.org/

Genome-wide Complex Trait Analysis (http://cnsgenomics.com/software/gcta/

Genotype Tissue Expression Project (https://www.gtexportal.org/home/)

Roadmap Epigenomics Consortium (http://www.roadmapepigenomics.org)

Encyclopedia of DNA Elements Project (https://www.encodeproject.org)

ChromHMM (http://compbio.mit.edu/ChromHMM/)

HaploReg (https://pubs.broadinstitute.org/mammals/haploreg/haploreg.php)

JASPAR (http://jaspar.genereg.net)

TRANSFAC–MATCH (http://gene-regulation.com/pub/programs.html)

HOCOMOCO (http://hocomoco11.autosome.ru)

ChIP-Cor (http://ccg.vital-it.ch/chipseq/chip_cor.php)

PWMScan (http://ccg.vital-it.ch/pwmtools/pwmscan.php)

MatrixEQTL (https://cran.r-project.org/web/packages/MatrixEQTL/index.html)

LocusZoom (http://locuszoom.org)

FINEMAP (http://www.christianbenner.com/#)

PAINTOR (https://github.com/gkichaev/PAINTOR_V3.0)

PICS (https://pubs.broadinstitute.org/pubs/finemapping/)

## Supporting information

S1 Fig rs2820315 is not associated with known CAD risk factors. LocusZoom plot depicting lack of genome-wide association of lead CAD SNP rs2820315 with known CAD risk factors including, (A) fasting glucose, (B) fasting insulin from Meta-Analysis of Glucose and Insulin-related traits Consortium (MAGIC), (C) Systolic blood pressure from ICBP-2011 and (D) low von Willebrand factor (VWF) from Cohorts for Heart and Aging Research in Genome Epidemiology (CHARGE) at chromosome 1. Circles represent SNPs associated using an additive or recessive model, and color-coded for LD (r^2^) with the lead SNP, rs2820315 (purple diamond), which resides near the *LMOD1* gene.
(PDF)

S2 Fig Lead CAD risk variant rs2820315 and LD variant rs34091558 are associated with *LMOD1* expression. (A and C) rs2820315 and (B and D) rs34091558 eQTL association dosage plots showing correlation of genotypes with *LMOD1* expression in lung and esophagus mucosa tissues in GTEx v6p dataset.
(PDF)

S3 Fig *LMOD1* is highly expressed in CAD related tissues. Expression levels of *LMOD1* and nearby genes in coronary, tibial and aorta artery tissues versus liver in (A) GTEx v6p (shown as RPKM) and in atherosclerotic aorta (AOR), mammary artery (MAM), and liver in (B) STARNET databases (shown as EDAseq normalized reads, as described in Methods).
(PDF)

S4 Fig Differential tissue expression pattern of neighboring genes at *LMOD1* locus. Tissue expression profile of (A) *IPO9 and* (B) *NAV1* neighboring genes across the entire GTEx v7 dataset ranked according to transcripts per million (TPM).
(PDF)

S5 Fig Haplotype analysis and allelic expression imbalance (AEI) of *LMOD1* in HCASMCs. (A) Linkage disequilibrium (LD) plot of the rs2820315 locus at 1q32.1 from 1000 genome phase 3 chromosome 1 haplotypes in Europeans, showing the lead SNP, rs2820315, in the same haploblock with a number of variants which are in high LD with rs34091558, including missense coding variant rs2820312. Red color-coded for LD based on r^2^ values, shown in boxes. (B) Log2 ratio of VIC/FAM intensity from HCASMCs homozygous for rs2820312 allele at cycle 50 generated by mixing DNA at the following ratios: 4:1, 2:1 1:1, 1:2, 1:4. A linear regression standard curve was generated to correct cDNA ratio by plotting against the Log ratio of the two alleles. (C) Representative pyrosequencing traces from HCASMC cDNA and gDNA from homozygous and heterozygous HCASMCs. Allelic ratios were quantitated from the area under the curve for both major and minor alleles using PyroMark Q24 software (Qiagen). Similar results were observed from n = 3 independent experiments.
(PDF)

S6 Fig Lead risk variant rs2820315 and LD variant rs34091558 reside in active enhancer regions. (A) WashU epigenomics browser screenshot showing overlap of rs2820315 and rs34091558 with ChIP-seq tracks for active enhancer histone modification H3K27ac found in different tissues. (B) UCSC genome browser screenshot revealing overlap of rs2820315 and rs34091558 in the ChIP-seq tracks for active enhancer histone modification H3K27ac present in different ENCODE cell lines.
(PDF)

S7 Fig rs34091558 resides in an active enhancer chromatin state. ChromHMM screenshot showing chromatin states of the risk variants found in the *LMOD1* gene in various tissue samples.
(PDF)

S8 Fig Conditional analysis at *LMOD1* locus using CAD GWAS summary statistics. Locus Zoom plot depicting the results of conditional testing of SNP rs34091558 using the latest CARDIoGRAMplusC4D and UK Biobank GWAS meta-analysis summary statistics data in GCTA-COJO. Purple diamond indicates the lead SNP rs2820315 signal. LD calculated using European population data.
(PDF)

S9 Fig *FOXO3* is enriched in CAD related and SMC enriched tissues. Expression profile of FOXO3 family members in (A) Aorta and (B) Tibial artery ranked according to RPKM in the GTEx dataset.
(PDF)

S10 Fig Validation of altered *FOXO3* expression following transfection. Quantitative RT-PCR analysis showing (A) reduced *FOXO3* expression in HCASMCs transfected with siRNA to *FOXO3* and (B) increased expression in A7r5 transfected with a plasmid encoding human *FOXO3*. (C) Western blot results showing reduced FOXO3 protein in HCASMC transfected with siRNA to FOXO3. TUBA represents beta-tubulin loading control.
(PDF)

S11 Fig TGFB1 does not regulate *LMOD1* expression. Quantitative RT-PCR analysis showing reduced *TGFB1* mRNA expression in cells transfected with siRNA to *TGFB1* (A) but no detectable changes in *LMOD1* mRNA expression levels (B).
(PDF)

S12 Fig FOXO3 motif density plots in HCASMC-specific open chromatin regions. (A) Motif density of FOXO3 position weight matrix (PWM) motifs centered on HCASMC-specific ATAC-seq regions of open chromatin, compared to open chromatin data from other tissues including Brain, Lung fibroblasts, and Heart. (B) FOXO3 PWM motif density in HCASMC-specific open chromatin peaks using the consensus motif or randomized motif to demonstrate specificity of the signal.
(PDF)

S1 Table eQTL associations for rs34091558 and rs2820315 in GTEx artery tissues. (PDF)

S2 Table PICS fine-mapping results of *LMOD1* CAD locus. (PDF)

S3 Table PAINTOR fine-mapping results of *LMOD1* CAD locus using HCASMC annotations. (PDF)

S4 Table FINEMAP fine-mapping results using GTEx eQTL and CAD summary results. (PDF)

S5 Table HaploReg and HCASMC functional annotations of *LMOD1* candidate regulatory variants. (PDF)

S6 Table Effects of rs34091558 SNP on TFBS predictions–JASPAR. (PDF)

S7 Table Primer/TaqMan Assay IDs. (PDF)
