# Supplementary figures and images for: Functional regulatory mechanism of smooth muscle cell-restricted LMOD1 coronary artery disease locus
Source: PLoS Genet. 2018 Nov 16;14(11):e1007755. doi: 10.1371/journal.pgen.1007755 (PMC6268002; doi:10.1371/journal.pgen.1007755)

**A** Fasting Glucose - MAGIC 2012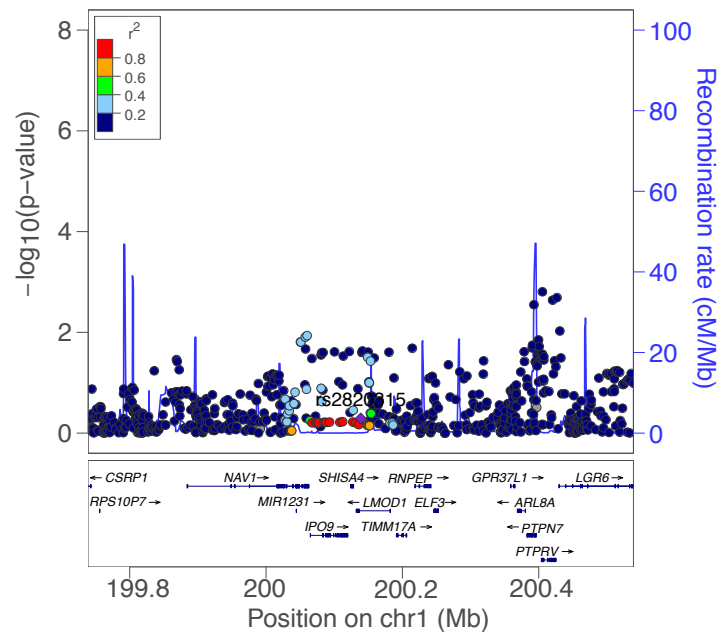**B** Fasting Insulin - MAGIC 2012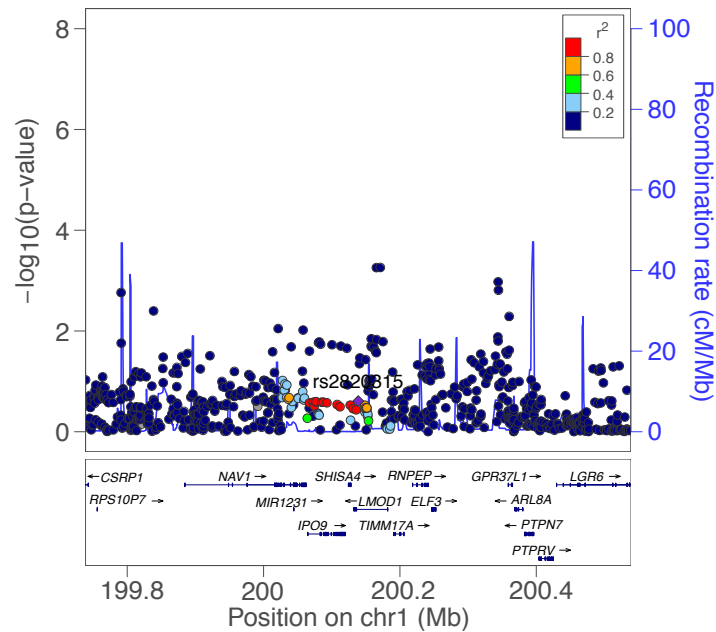**C** Systolic Blood Pressure - ICBP 2011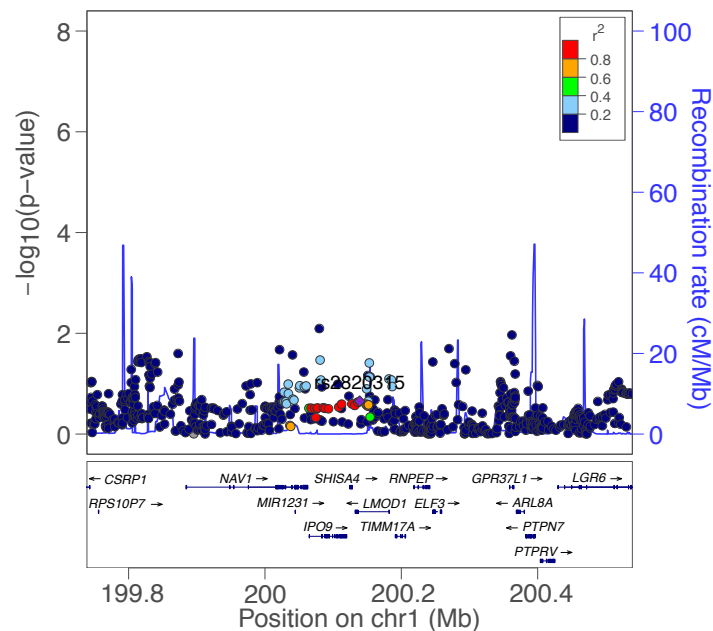**D** Low vWF - CHARGE 2014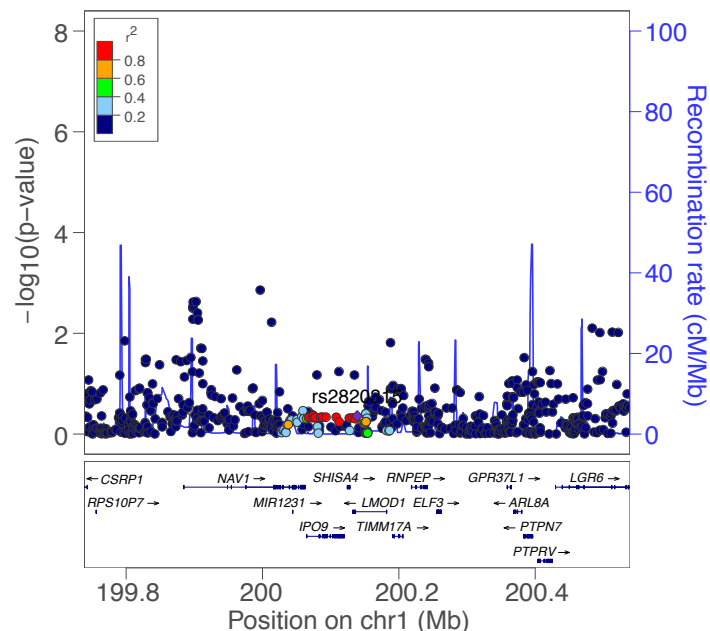

Supplement: S1 Fig — LocusZoom plot depicting lack of genome-wide association of lead CAD SNP rs2820315 with known CAD risk factors including, (A) fasting glucose, (B) fasting insulin from Meta-Analysis of Glucose and Insulin-related traits Consortium (MAGIC), (C) Systolic blood pressure from ICBP-2011 and (D) low von Willebrand factor (VWF) from Cohorts for Heart and Aging Research in Genome Epidemiology (CHARGE) at chromosome 1. Circles represent SNPs associated using an additive or recessive model, and color-coded for LD (r2) with the lead SNP, rs2820315 (purple diamond), which resides near the LMOD1 gene. (PDF) [file pgen.1007755.s001.pdf]

**A**

## GTEx tissue gene expression

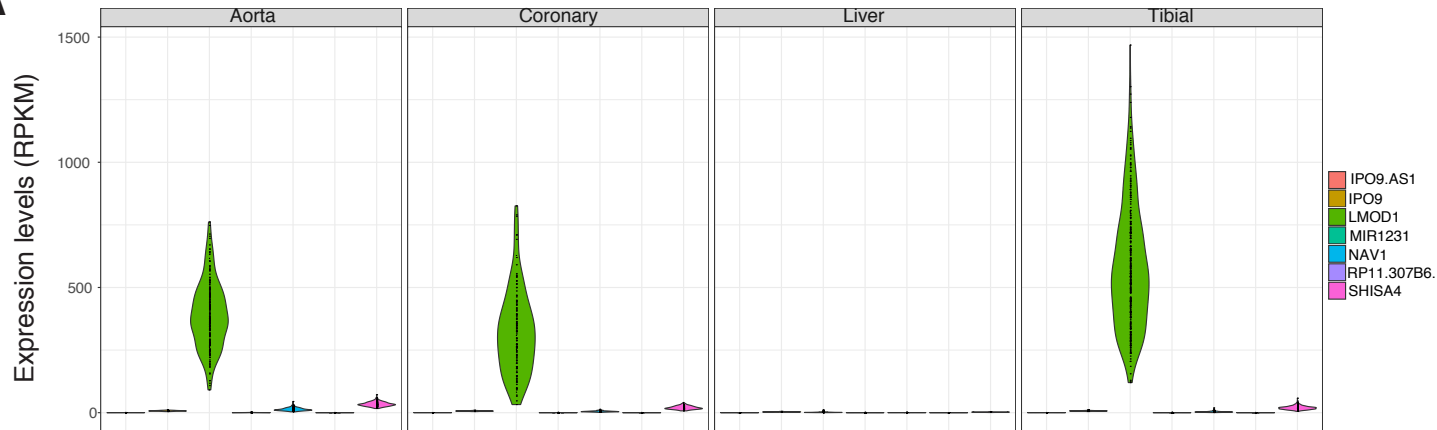**B**

## STARNET tissue gene expression

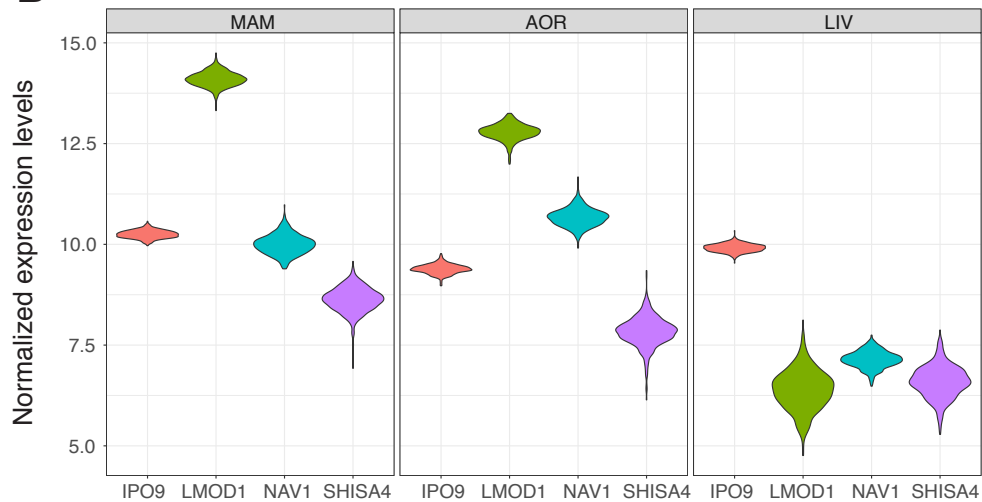

Supplement: S3 Fig — Expression levels of LMOD1 and nearby genes in coronary, tibial and aorta artery tissues versus liver in (A) GTEx v6p (shown as RPKM) and in atherosclerotic aorta (AOR), mammary artery (MAM), and liver in (B) STARNET databases (shown as EDAseq normalized reads, as described in Methods). (PDF) [file pgen.1007755.s003.pdf]

**A***IPO9*

GTEx Tissue

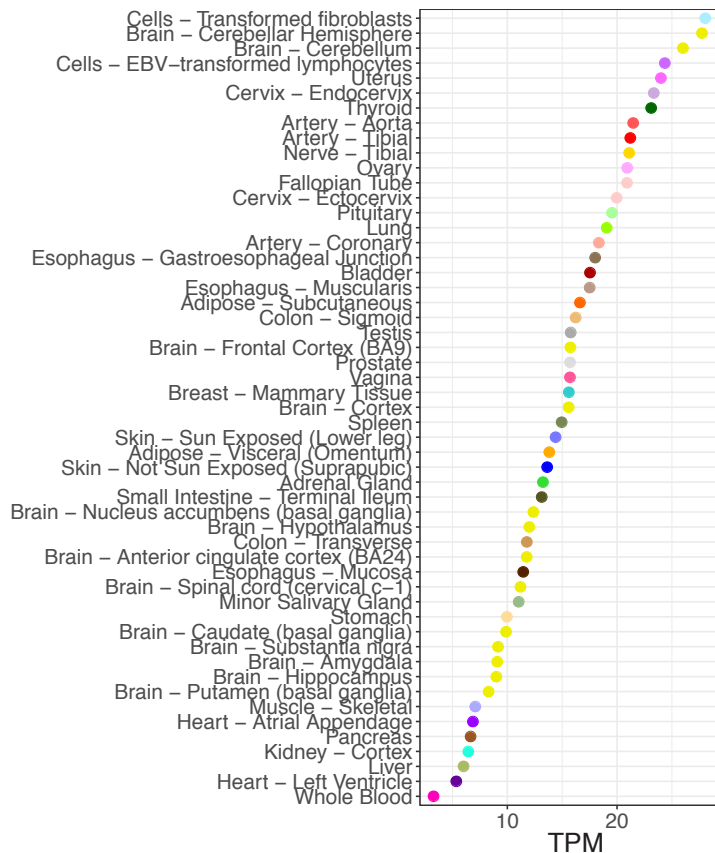**B***NAV1*

GTEx Tissue

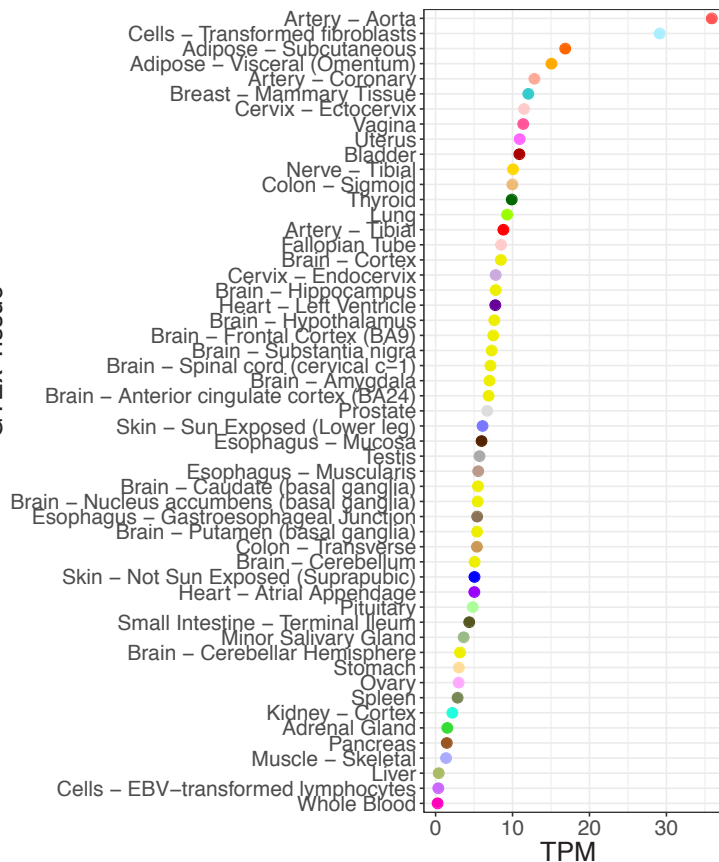

Supplement: S4 Fig — Tissue expression profile of (A) IPO9 and (B) NAV1 neighboring genes across the entire GTEx v7 dataset ranked according to transcripts per million (TPM). (PDF) [file pgen.1007755.s004.pdf]

A

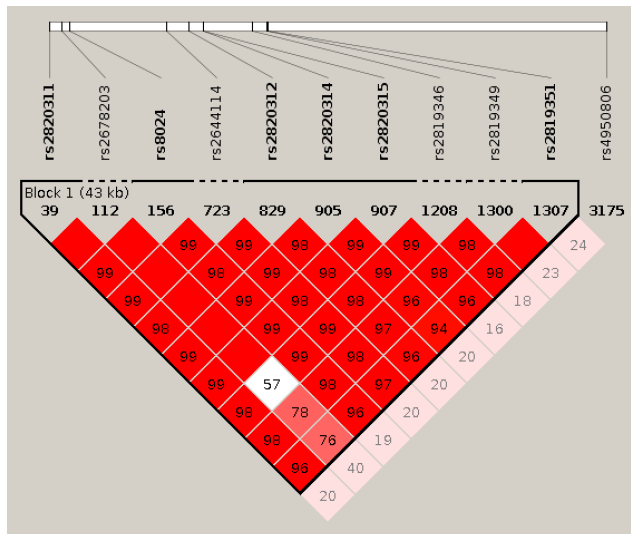

B

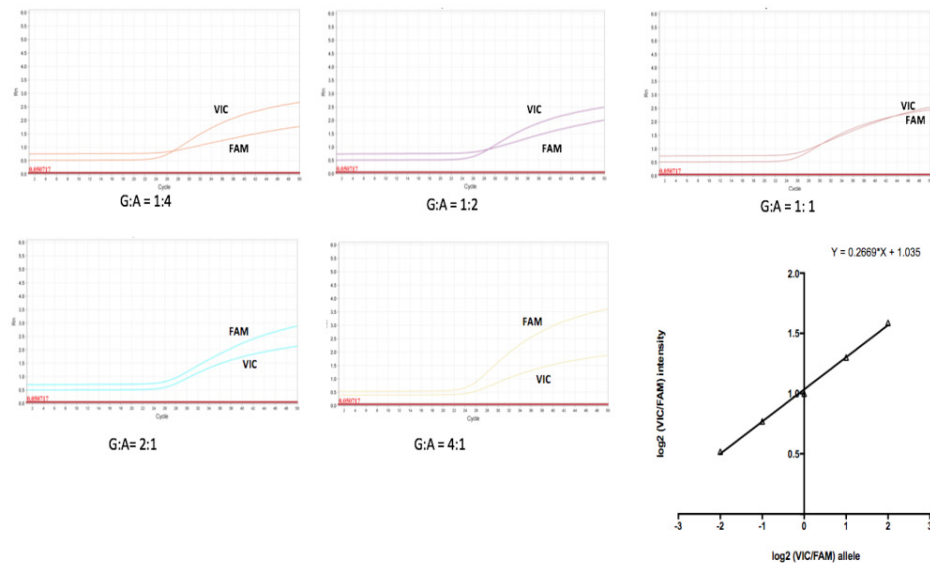

C

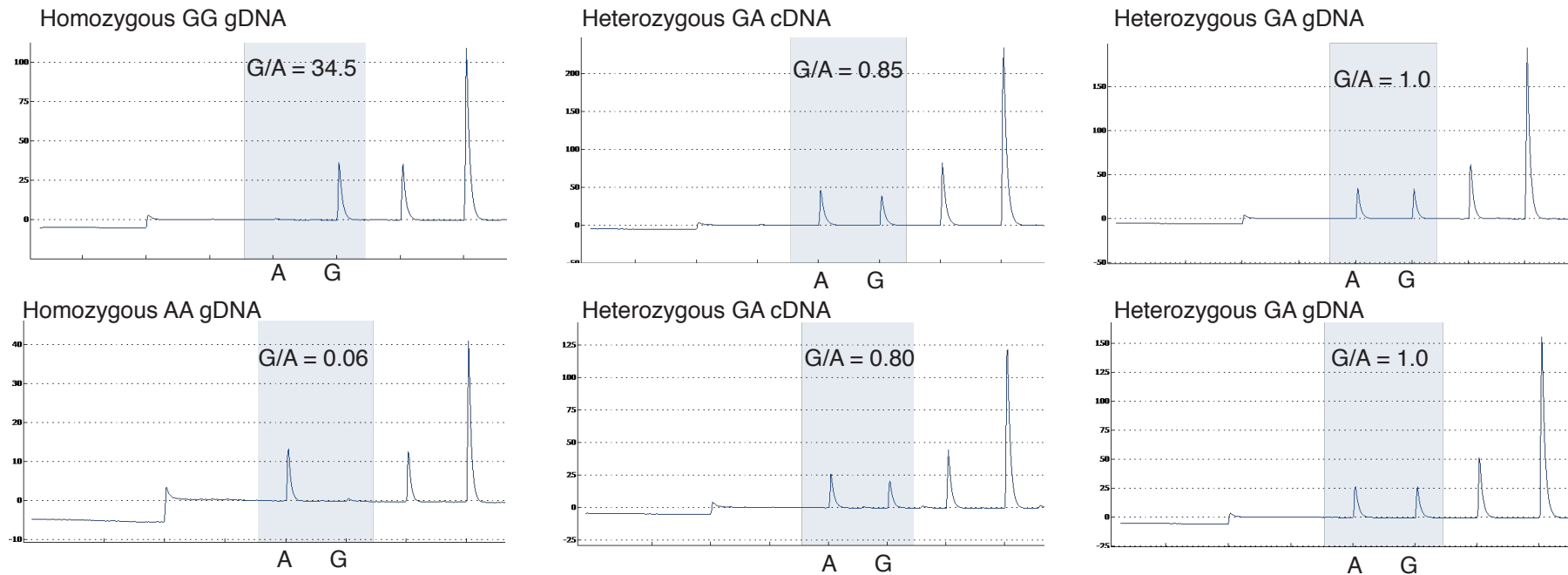

Supplement: S5 Fig — (A) Linkage disequilibrium (LD) plot of the rs2820315 locus at 1q32.1 from 1000 genome phase 3 chromosome 1 haplotypes in Europeans, showing the lead SNP, rs2820315, in the same haploblock with a number of variants which are in high LD with rs34091558, including missense coding variant rs2820312. Red color-coded for LD based on r2 values, shown in boxes. (B) Log2 ratio of VIC/FAM intensity from HCASMCs homozygous for rs2820312 allele at cycle 50 generated by mixing DNA at the following ratios: 4:1, 2:1 1:1, 1:2, 1:4. A linear regression standard curve was generated to correct cDNA ratio by plotting against the Log ratio of the two alleles. (C) Representative pyrosequencing traces from HCASMC cDNA and gDNA from homozygous and heterozygous HCASMCs. Allelic ratios were quantitated from the area under the curve for both major and minor alleles using PyroMark Q24 software (Qiagen). Similar results were observed from n = 3 independent experiments. (PDF) [file pgen.1007755.s005.pdf]

**A**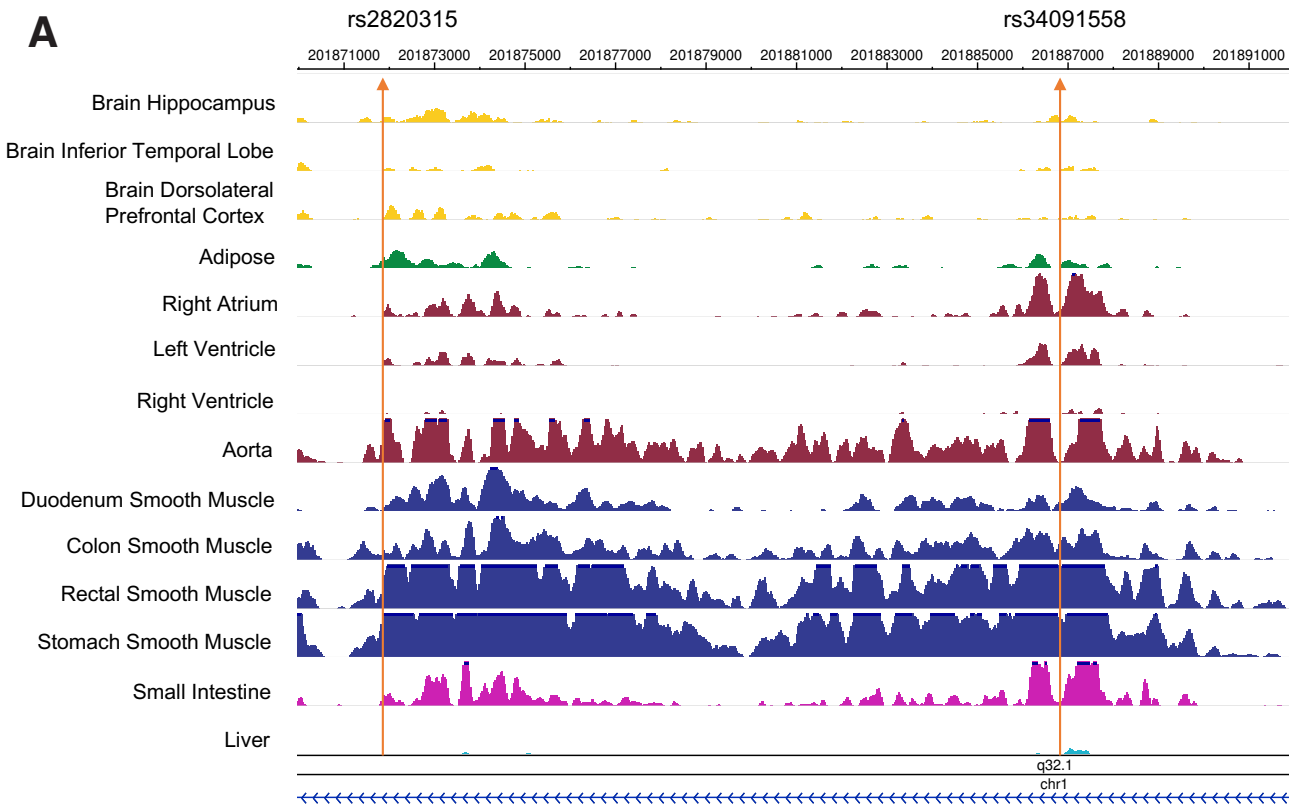**B**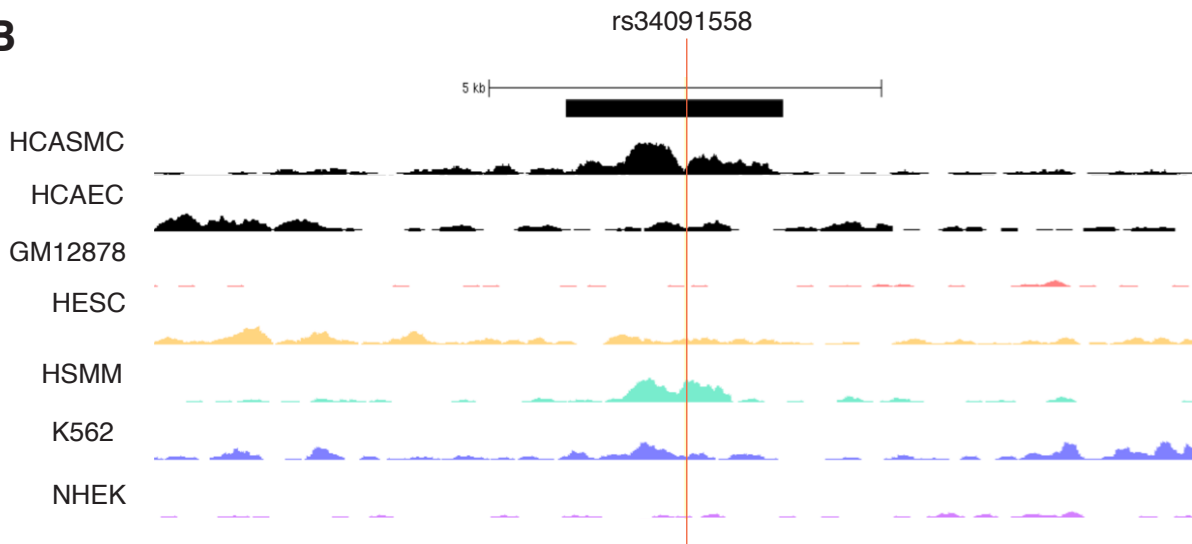

Supplement: S6 Fig — (A) WashU epigenomics browser screenshot showing overlap of rs2820315 and rs34091558 with ChIP-seq tracks for active enhancer histone modification H3K27ac found in different tissues. (B) UCSC genome browser screenshot revealing overlap of rs2820315 and rs34091558 in the ChIP-seq tracks for active enhancer histone modification H3K27ac present in different ENCODE cell lines. (PDF) [file pgen.1007755.s006.pdf]

# Conditional analysis on rs34091558 in CARDIoGRAMplus C4D + UK Biobank

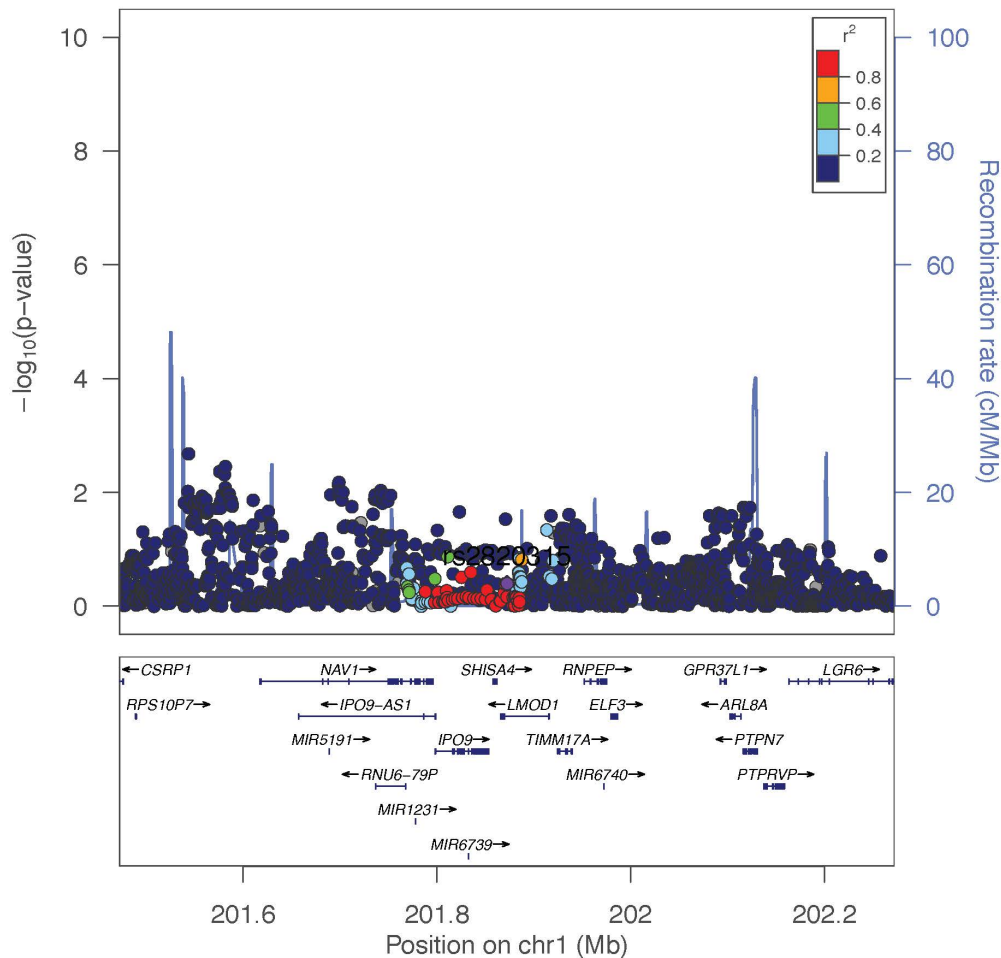

Supplement: S8 Fig — Locus Zoom plot depicting the results of conditional testing of SNP rs34091558 using the latest CARDIoGRAMplusC4D and UK Biobank GWAS meta-analysis summary statistics data in GCTA-COJO. Purple diamond indicates the lead SNP rs2820315 signal. LD calculated using European population data. (PDF) [file pgen.1007755.s008.pdf]

**A**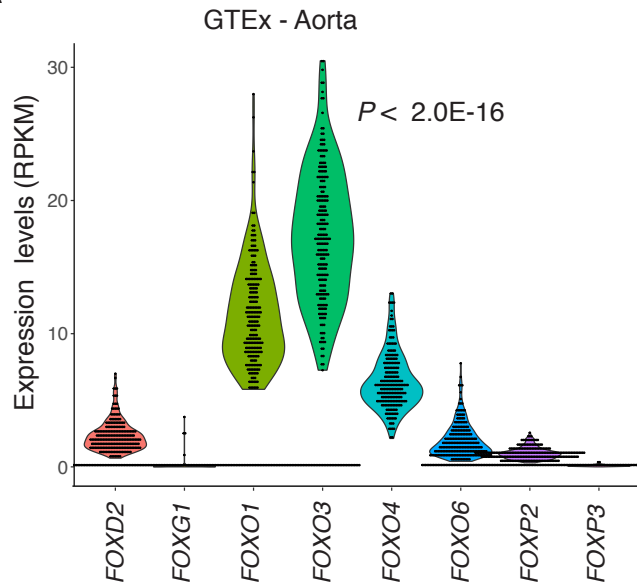**B**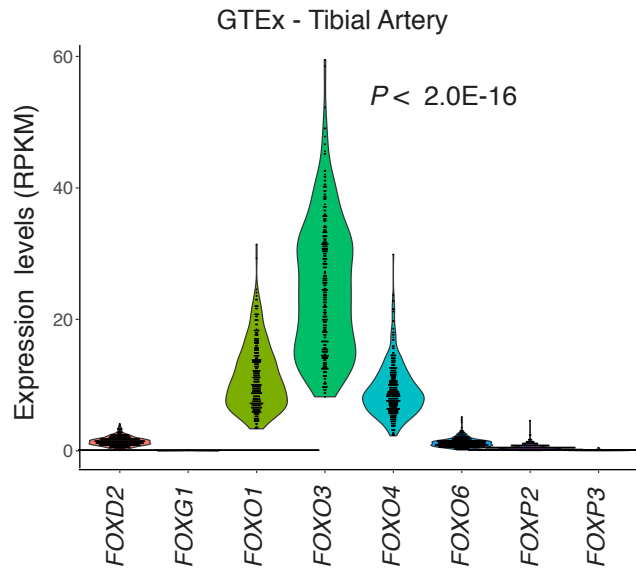

Supplement: S9 Fig — Expression profile of FOXO3 family members in (A) Aorta and (B) Tibial artery ranked according to RPKM in the GTEx dataset. (PDF) [file pgen.1007755.s009.pdf]

**A**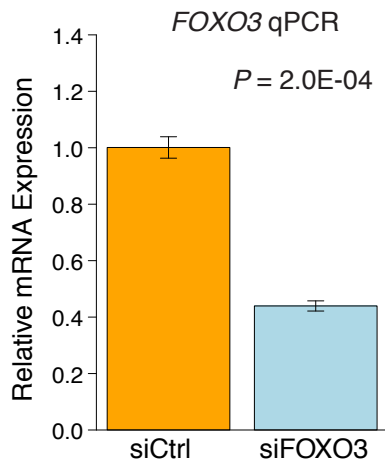**B**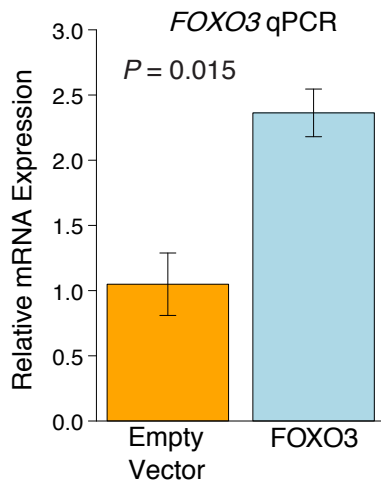**C**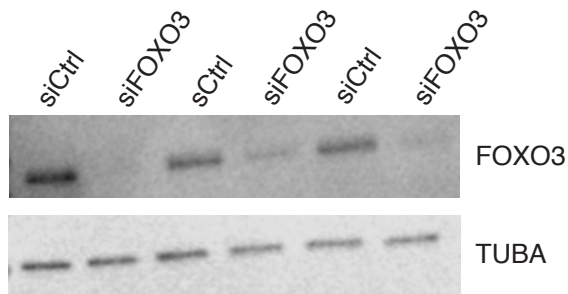

Supplement: S10 Fig — Quantitative RT-PCR analysis showing (A) reduced FOXO3 expression in HCASMCs transfected with siRNA to FOXO3 and (B) increased expression in A7r5 transfected with a plasmid encoding human FOXO3. (C) Western blot results showing reduced FOXO3 protein in HCASMC transfected with siRNA to FOXO3. TUBA represents beta-tubulin loading control. (PDF) [file pgen.1007755.s010.pdf]

**A**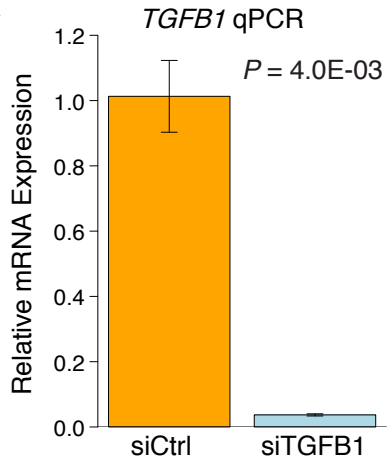**B**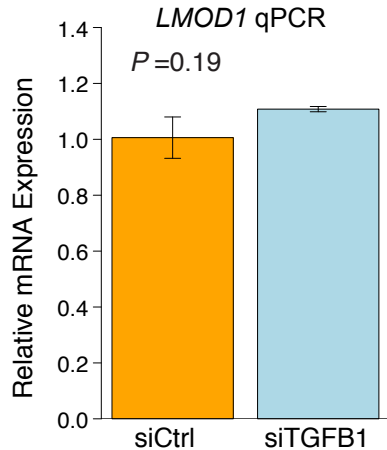

Supplement: S11 Fig — Quantitative RT-PCR analysis showing reduced TGFB1 mRNA expression in cells transfected with siRNA to TGFB1 (A) but no detectable changes in LMOD1 mRNA expression levels (B). (PDF) [file pgen.1007755.s011.pdf]

**A**

Density of FOXO3 PWM sites

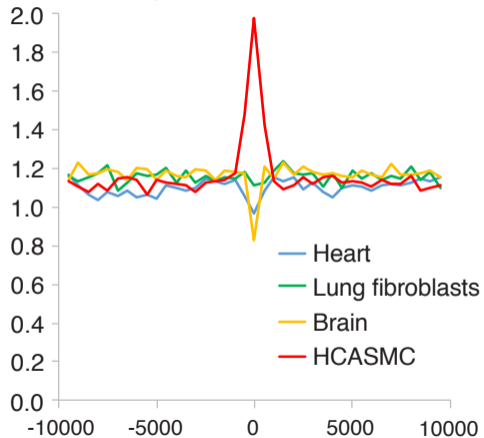**B**

Density of FOXO3 PWM sites

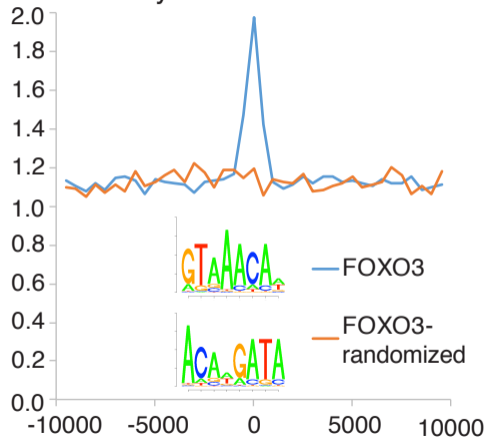

Supplement: S12 Fig — (A) Motif density of FOXO3 position weight matrix (PWM) motifs centered on HCASMC-specific ATAC-seq regions of open chromatin, compared to open chromatin data from other tissues including Brain, Lung fibroblasts, and Heart. (B) FOXO3 PWM motif density in HCASMC-specific open chromatin peaks using the consensus motif or randomized motif to demonstrate specificity of the signal. (PDF) [file pgen.1007755.s012.pdf]
