# Supplementary material for: Functional regulatory mechanism of smooth muscle cell-restricted LMOD1 coronary artery disease locus
Source: PLoS Genet. 2018 Nov 16;14(11):e1007755. doi: 10.1371/journal.pgen.1007755 (PMC6268002; doi:10.1371/journal.pgen.1007755)
Supplement: S2 Fig — (A and C) rs2820315 and (B and D) rs34091558 eQTL association dosage plots showing correlation of genotypes with LMOD1 expression in lung and esophagus mucosa tissues in GTEx v6p dataset. (PDF) [file pgen.1007755.s002.pdf]

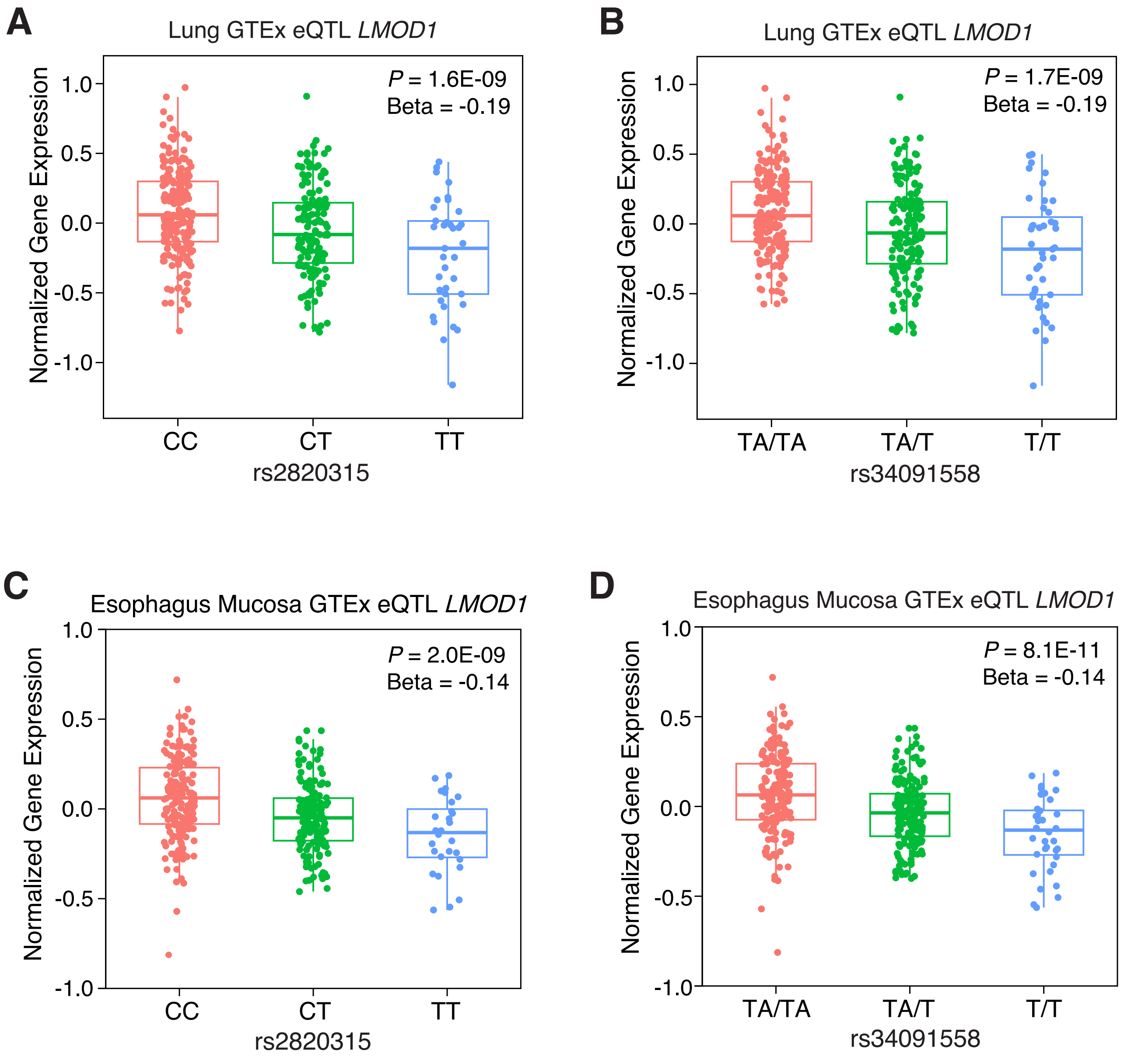
A
Lung GTEx eQTL LMOD1
P = 1.6E-09
Beta = -0.19
Normalized Gene Expression
1.0
0.5
0.0
-0.5
-1.0
CC
CT
TT
rs2820315
B
Lung GTEx eQTL LMOD1
P = 1.7E-09
Beta = -0.19
Normalized Gene Expression
TA/TA
TA/T
T/T
rs34091558
C
Esophagus Mucosa GTEx eQTL LMOD1
P = 2.0E-09
Beta = -0.14
Normalized Gene Expression
CC
CT
TT
rs2820315
D
Esophagus Mucosa GTEx eQTL LMOD1
P = 8.1E-11
Beta = -0.14
Normalized Gene Expression
TA/TA
TA/T
T/T
rs34091558
