# Supplementary material for: Functional regulatory mechanism of smooth muscle cell-restricted LMOD1 coronary artery disease locus
Source: PLoS Genet. 2018 Nov 16;14(11):e1007755. doi: 10.1371/journal.pgen.1007755 (PMC6268002; doi:10.1371/journal.pgen.1007755)
Supplement: S7 Fig — ChromHMM screenshot showing chromatin states of the risk variants found in the LMOD1 gene in various tissue samples. (PDF) [file pgen.1007755.s007.pdf]

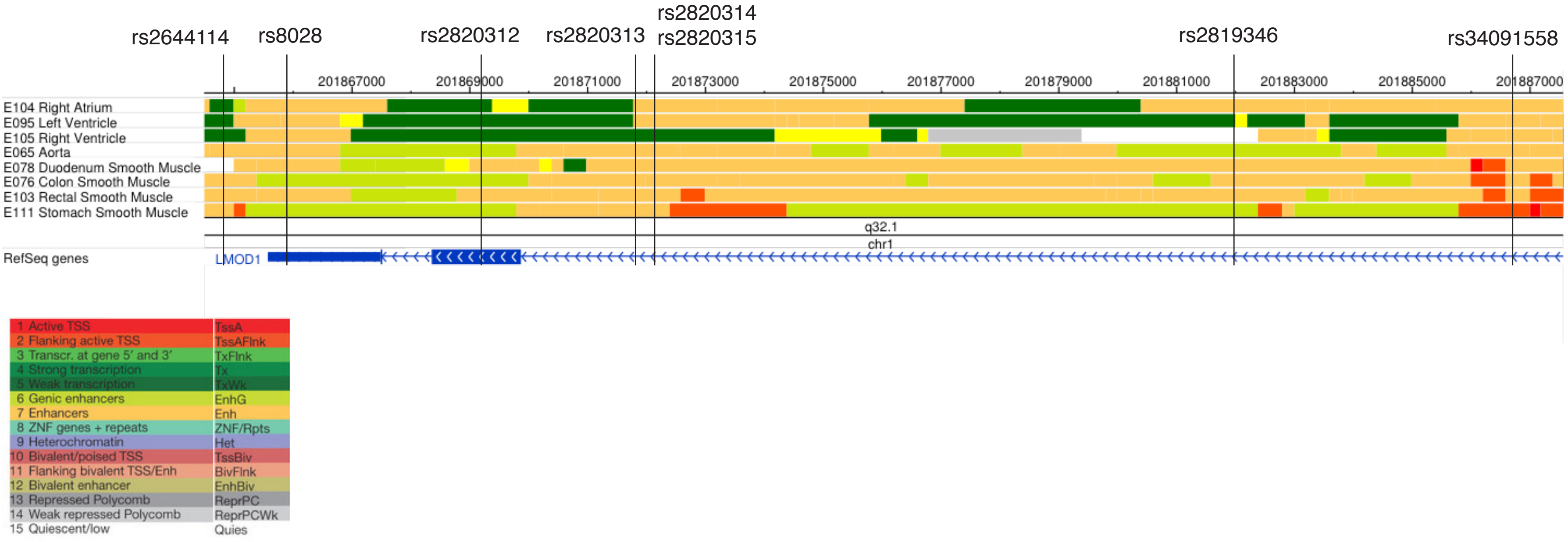
rs2644114
rs8028
rs2820312
rs2820313
rs2820314
rs2820315
rs2819346
rs34091558
201867000
201869000
201871000
201873000
201875000
201877000
201879000
201881000
201883000
201885000
201887000
E104 Right Atrium
E095 Left Ventricle
E105 Right Ventricle
E065 Aorta
E078 Duodenum Smooth Muscle
E076 Colon Smooth Muscle
E103 Rectal Smooth Muscle
E111 Stomach Smooth Muscle
q32.1
chr1
RefSeq genes
LMOD1
1 Active TSS | TssA
2 Flanking active TSS | TssAFlnk
3 Transcr. at gene 5' and 3' | TxFlnk
4 Strong transcription | Tx
5 Weak transcription | TxWk
6 Genic enhancers | EnhG
7 Enhancers | Enh
8 ZNF genes + repeats | ZNF/Rpts
9 Heterochromatin | Het
10 Bivalent/poised TSS | TssBiv
11 Flanking bivalent TSS/Enh | BivFlnk
12 Bivalent enhancer | EnhBiv
13 Repressed Polycomb | ReprPC
14 Weak repressed Polycomb | ReprPCWk
15 Quiescent/low | Quies
