# Supplementary material for: Functional regulatory mechanism of smooth muscle cell-restricted LMOD1 coronary artery disease locus
Source: PLoS Genet. 2018 Nov 16;14(11):e1007755. doi: 10.1371/journal.pgen.1007755 (PMC6268002; doi:10.1371/journal.pgen.1007755)
Supplement: S1 Table — (PDF) [file pgen.1007755.s013.pdf]

S1 Table. eQTL associations for rs34091558 and rs2820315 in GTEx artery tissues.

GTEx Tissue	Ensembl ID	Gene Symbol	eSNP	Nominal P-value	Effect size (beta)
Tibial Artery	ENSG00000163431	***LMOD1***	rs34091558	**4.99E-09**	-0.1133
Tibial Artery	ENSG00000198892	*SHISA4*	rs34091558	0.1622	-0.0357
Tibial Artery	ENSG00000198700	*IPO9*	rs34091558	0.0104	0.0778
Tibial Artery	ENSG00000231871	*IPO9-AS1*	rs34091558	0.9056	-0.0096
Tibial Artery	ENSG00000134369	*NAV1*	rs34091558	0.1043	0.0518
Tibial Artery	ENSG00000163431	***LMOD1***	rs2820315	**1.24E-07**	-0.1055
Tibial Artery	ENSG00000198892	*SHISA4*	rs2820315	0.0588	-0.0495
Tibial Artery	ENSG00000198700	*IPO9*	rs2820315	0.0364	0.0654
Tibial Artery	ENSG00000231871	*IPO9-AS1*	rs2820315	0.9378	-0.0065
Tibial Artery	ENSG00000134369	*NAV1*	rs2820315	0.1482	0.0474
Coronary Artery	ENSG00000163431	*LMOD1*	rs34091558	0.0157	-0.0914
Coronary Artery	ENSG00000198892	*SHISA4*	rs34091558	0.6137	0.0266
Coronary Artery	ENSG00000198700	*IPO9*	rs34091558	0.1969	0.0596
Coronary Artery	ENSG00000231871	*IPO9-AS1*	rs34091558	0.221	0.1444
Coronary Artery	ENSG00000134369	*NAV1*	rs34091558	0.2022	0.1186
Coronary Artery	ENSG00000163431	*LMOD1*	rs2820315	0.0132	-0.0971
Coronary Artery	ENSG00000198892	*SHISA4*	rs2820315	0.8806	0.0082
Coronary Artery	ENSG00000198700	*IPO9*	rs2820315	0.1256	0.0732
Coronary Artery	ENSG00000231871	*IPO9-AS1*	rs2820315	0.186	0.1615
Coronary Artery	ENSG00000134369	*NAV1*	rs2820315	0.0723	0.1725
Aorta Artery	ENSG00000163431	*LMOD1*	rs34091558	0.0157	-0.0914
Aorta Artery	ENSG00000198892	*SHISA4*	rs34091558	0.953	-0.0021
Aorta Artery	ENSG00000198700	*IPO9*	rs34091558	0.0003	0.1191
Aorta Artery	ENSG00000231871	*IPO9-AS1*	rs34091558	0.0443	0.1321
Aorta Artery	ENSG00000134369	*NAV1*	rs34091558	0.1883	0.0537
Aorta Artery	ENSG00000163431	*LMOD1*	rs2820315	0.0011	-0.1088
Aorta Artery	ENSG00000198892	*SHISA4*	rs2820315	0.6838	0.016
Aorta Artery	ENSG00000198700	*IPO9*	rs2820315	0.0001	0.1351
Aorta Artery	ENSG00000231871	*IPO9-AS1*	rs2820315	0.0399	0.1425
Aorta Artery	ENSG00000134369	*NAV1*	rs2820315	0.1049	0.0698
