# Supplementary material for: Functional regulatory mechanism of smooth muscle cell-restricted LMOD1 coronary artery disease locus
Source: PLoS Genet. 2018 Nov 16;14(11):e1007755. doi: 10.1371/journal.pgen.1007755 (PMC6268002; doi:10.1371/journal.pgen.1007755)
Supplement: S2 Table — (PDF) [file pgen.1007755.s014.pdf]

S2 Table. PICS fine-mapping results of *LMOD1* CAD locus.

Index SNP	Linked SNP	Dprime	R-Square	Phase	PICS_probability
rs2820315	rs2820315	1	1	N,N	0.1579
rs2820315	rs34091558	0.9937	0.9399	C,R	0.0419
rs2820315	rs2820314	1	0.929	C,A	0.0368
rs2820315	rs2820323	1	0.896	C,G	0.0253
rs2820315	rs2820321	1	0.896	C,G	0.0253
rs2820315	rs2820319	1	0.896	C,G	0.0253
rs2820315	rs2819351	1	0.896	C,C	0.0253
rs2820315	rs2819349	1	0.896	C,C	0.0253
rs2820315	rs2819347	1	0.896	C,C	0.0253
rs2820315	rs2820322	1	0.8907	C,T	0.0239
rs2820315	rs2820318	1	0.8907	C,T	0.0239
rs2820315	rs2820317	1	0.8907	C,G	0.0239
rs2820315	rs2819348	1	0.8907	C,T	0.0239
rs2820315	rs2819346	1	0.8907	C,A	0.0239
rs2820315	rs2644121	1	0.8854	C,A	0.0226
rs2820315	rs201603952	1	0.8495	C,R	0.0155
rs2820315	rs5780094	0.9191	0.8447	C,R	0.0147
rs2820315	rs72310529	1	0.8445	C,R	0.0147
rs2820315	rs2820310	0.9429	0.836	C,G	0.0135
rs2820315	rs2494115	0.9429	0.836	C,G	0.0135
rs2820315	rs2254614	0.9429	0.836	C,T	0.0135
rs2820315	rs2644134	0.9247	0.8342	C,G	0.0132
rs2820315	rs8028	0.9428	0.8307	C,G	0.0127
rs2820315	rs2820312	0.9428	0.8307	C,G	0.0127
rs2820315	rs2820311	0.9428	0.8307	C,A	0.0127
