# Supplementary material for: Functional regulatory mechanism of smooth muscle cell-restricted LMOD1 coronary artery disease locus
Source: PLoS Genet. 2018 Nov 16;14(11):e1007755. doi: 10.1371/journal.pgen.1007755 (PMC6268002; doi:10.1371/journal.pgen.1007755)
Supplement: S3 Table — (PDF) [file pgen.1007755.s015.pdf]

S3 Table. PAINTOR fine-mapping results of *LMOD1* CAD locus using HCASMC annotations.

chr	pos	rsid	ref	alt	Zscore	Posterior_Prob
chr1	201886769	rs34091558	TA	T	-4.23147	1
chr1	201500825	rs4915537	T	C	-1.21108	0.991236
chr1	201885157	rs2819351	T	C	3.93095	0.991236
chr1	201907945	rs7533340	C	T	-1.01796	0.991236
chr1	201805926	rs16849383	C	T	-0.991702	0.00876378
chr1	201884952	rs2819348	C	T	3.80651	0.00876378
chr1	201872264	rs2820315	T	C	4.52362	2.18E-19
chr1	201883160	rs2820323	A	G	3.74832	2.18E-19
chr1	201882087	rs2819346	C	A	3.64641	2.63E-23
chr1	201802052	rs2644111	A	G	3.85847	3.09E-27
chr1	201880300	rs2820322	C	T	3.62866	3.09E-27
chr1	201851492	rs2644134	A	G	-4.29502	3.05E-28
chr1	201872209	rs2820314	C	A	4.02326	1.72E-28
chr1	201881284	rs2644121	G	A	3.63377	1.72E-28
chr1	201885026	rs2819349	T	C	3.91618	1.72E-28
chr1	202067723	rs4245702	G	A	-1.16991	1.72E-28
chr1	201787940	rs2678210	C	T	4.35153	1.25E-33
chr1	201821707	rs74430074	G	A	-1.00873	1.25E-33
chr1	201825750	rs10800793	G	A	-1.71147	1.25E-33
chr1	202053119	rs143250450	A	G	1.62979	6.76E-35
chr1	201520113	rs116349281	T	C	1.3849	3.03E-38
chr1	201712172	rs12089993	T	C	-2.09661	3.03E-38
chr1	201825277	rs56923474	T	C	4.0542	3.03E-38
chr1	201654790	rs1414972	T	C	-1.32103	8.23E-42
chr1	201956217	rs6690438	T	C	-0.861696	8.23E-42
