# Supplementary material for: Functional regulatory mechanism of smooth muscle cell-restricted LMOD1 coronary artery disease locus
Source: PLoS Genet. 2018 Nov 16;14(11):e1007755. doi: 10.1371/journal.pgen.1007755 (PMC6268002; doi:10.1371/journal.pgen.1007755)
Supplement: S4 Table — (PDF) [file pgen.1007755.s016.pdf]

S4 Table. FINEMAP fine-mapping results using GTEx eQTL and CAD summary results.

index	snp	rsid	snp_prob	snp_log10bf
812	201886769	rs34091558	0.9274	4.174
801	201884952	rs2819348	0.0396	1.6825
789	201881284	rs2644121	0.0047	0.7451
788	201880300	rs2820322	0.0047	0.7451
783	201878620	rs2820318	0.0047	0.7451
782	201878537	rs2820317	0.0047	0.7451
787	201880296	rs2820321	0.0042	0.6925
784	201878650	rs2820319	0.0042	0.6925
796	201884288	rs2819347	0.0027	0.4988
803	201885157	rs2819351	0.0008	-0.0486
790	201882087	rs2819346	0.0007	-0.0582
802	201885026	rs2819349	0.0006	-0.1726
773	201872264	rs2820315	0.0002	-0.6499
768	201869257	rs2820312	0.0001	-0.8948
769	201870221	rs2820313	0.0001	-1.0002
765	201865763	rs8028	0.0001	-1.0501
717	201830247	rs2254614	0	-1.3467
671	201800511	rs2678204	0	-1.3476
745	201850057	rs1517810	0	-1.3777
710	201826482	rs2132363	0	-1.3777
702	201821443	rs1400875	0	-1.4242
772	201872209	rs2820314	0	-1.4242
687	201809918	rs903678	0	-1.4945
758	201860626	rs2250377	0	-1.4981
720	201832864	rs2494115	0	-1.5285
