# Supplementary material for: Functional regulatory mechanism of smooth muscle cell-restricted LMOD1 coronary artery disease locus
Source: PLoS Genet. 2018 Nov 16;14(11):e1007755. doi: 10.1371/journal.pgen.1007755 (PMC6268002; doi:10.1371/journal.pgen.1007755)
Supplement: S5 Table — (PDF) [file pgen.1007755.s017.pdf]

S5 Table. HaploReg and HCASMC functional annotations of *LMOD1* candidate regulatory variants.

Chr	Pos (hg19)	SNP	ref	alt	AFR freq	AMR freq	ASN freq	EUR freq	GERP cons	SIPhy cons	Gene Annotation	HCASMC annotations	TF motifs altered
1	201864830	rs2644114	A	G	0.75	0.75	0.89	0.68	No	No	None	-	CCNT2, GATA,HDAC2,HMGN3, TAL1
1	201865763	rs8028	A	G	0.74	0.75	0.89	0.68	No	No	3’-UTR	H3K4me1, H3K4me3, H3K27ac	PBX3, HIC1
1	201869257	rs2820312	G	A	0.27	0.25	0.11	0.32	No	No	missense	-	TAL1
1	201870221	rs2820313	A	G	0.4	0.26	0.11	0.32	No	No	intronic	-	BDP1
1	201872209	rs2820314	A	C	0.28	0.25	0.11	0.32	No	No	intronic	H3K4me1, H3K27ac, ATAC	EWSR1-FLI1, IRF, STAT
1	201872264	rs2820315	A	C	0.08	0.23	0.11	0.3	No	No	intronic	H3K4me1, H3K27ac, ATAC	-
1	201882087	rs2819346	A	C	0.41	0.26	0.11	0.33	No	No	intronic	H3K4me1	-
1	201882087	rs34091558	TA	T	0.16	0.24	0.12	0.32	Yes	Yes	intronic	H3K4me1, H3K4me3, H3K27ac, ATAC	FOXO1, FOXO2, FOXO3, FOXF2, GATA, GR
