# Supplementary material for: Functional regulatory mechanism of smooth muscle cell-restricted LMOD1 coronary artery disease locus
Source: PLoS Genet. 2018 Nov 16;14(11):e1007755. doi: 10.1371/journal.pgen.1007755 (PMC6268002; doi:10.1371/journal.pgen.1007755)
Supplement: S6 Table — (PDF) [file pgen.1007755.s018.pdf]

S6 Table. Effects of rs34091558 SNP on TFBS predictions – JASPAR.

TF	Relative score with non-risk allele(TA)	Relative score with risk allele (T)	Start	End	Strand	Predicted site sequence
FOXO3	0.912811839	0.6712661997	24	31	1	ATaAACAA
Foxj2	0.995583773	0.599554113	24	31	1	ATaAACAA
FOXL1	0.993644848	0.70792828	24	30	1	ATaAACA
FOXG1	0.986271908	0.601294322	24	31	1	ATaAACAA
FOXP3	0.978500686	0.651449584	24	30	1	ATaAACA
FOXD2	0.9778315	0.658205573	24	30	1	ATaAACA
FOXO4	0.966856102	0.699013536	24	30	1	ATaAACA
FOXO6	0.961235393	0.666238404	24	30	1	ATaAACA
GATA3	0.947950121	0.880039471	22	29	1	AGATaAAC
FOXI1	0.942137921	0.681867373	24	30	1	ATaAACA
GATA5	0.937937861	0.835939939	22	29	1	AGATaAAC
FOXP2	0.932396163	0.550531421	22	32	1	AGATaAACAAG
Gata4	0.932341169	0.960161265	19	29	-1	GTTtATCTCCC
Gata1	0.931110197	0.917970273	20	30	-1	TGTTtATCTCC
Foxo1	0.921336839	0.814379019	23	33	-1	ACTTGTTtATC
GATA2	0.90370878	0.930485573	20	33	-1	ACTTGTTtATCTCC
