# Supplementary material for: Functional regulatory mechanism of smooth muscle cell-restricted LMOD1 coronary artery disease locus
Source: PLoS Genet. 2018 Nov 16;14(11):e1007755. doi: 10.1371/journal.pgen.1007755 (PMC6268002; doi:10.1371/journal.pgen.1007755)
Supplement: S7 Table — (PDF) [file pgen.1007755.s019.pdf]

S7 Table. Primer/TaqMan Assay IDs.

Gene	Primer Sequence/TaqMan Probe Assay ID
5′-biotinylated forward rs2820312	GGGTCATTGTTCTTCACTCTCTCC
5′-biotinylated reverse rs2820312	AGACCAAAACACCCGAGAAAC
Reverse pyrosequencing primer	CACCCGAGAAACAGA
LMOD1	Hs00201704_m1
FOXO3	Hs00921424_m1
TGFB1	Hs00998133_m1
Lmod1	Rn01483340_m1
Fwd-h-Foxo3_v1	GATAC GATATC GCAGAGGCACCGGCTTCCCCGGCCCCG
Rev-h-Foxo3_v1	GATAC AAGCTT TCAGCCTGGCACCCAGCTCTGAGATGAGG
Fwd-LMOD1-ChIP	AGTACTAGCCAGGCACTTCA
Rev-LMOD1-ChIP	GGAGAAACCGGGAAATCTCTTT
Fwd-EGFR-ChIP	TCTGTGGCTGACTCCATCTG
Rev-EGFR-ChIP	CCCGTGACTCACTCCTTGAT
